# The native conformational landscape and priming mechanism of herpes simplex virus glycoprotein B

**DOI:** 10.1126/sciadv.aed8023

**Published:** 2026-07-24

**Authors:** Zongjun Mou, Shanshan Wang, Lauren Swanback, Yong Pan, Tiffany Tsai, Peicheng Ji, Jordan Su, Bibekananda Sahoo, Xinghong Dai

**Affiliations:** Department of Physiology and Biophysics, Case Western Reserve University School of Medicine, Cleveland, OH 44106, USA.

## Abstract

Glycoprotein B (gB) of herpesviruses mediates membrane fusion with host cells during viral entry. Stabilizing gB in its prefusion conformation is a primary strategy for vaccine development. While prefusion-like gB structures of several human herpesviruses have been solved, the native conformational landscape and structural dynamics of gB remain largely unknown. Here, we report cryo–electron microscopy structures of herpes simplex virus type 1 (HSV-1) gB from virions, revealing a predominant prefusion state and a minor population of an intermediate, primed state. Unique to α-herpesviruses, a tethering helix cross-links adjacent protomers and stabilizes these conformations. A further downstream intermediate we named as the deep-primed state was captured in a mutant and showed that structural changes in the central helices drive the disengagement of the fusion loops from the membrane-proximal regions, priming gB for membrane insertion. Leveraging these structural insights, we engineered gB mutants locked in distinct conformational states. Our findings provide an atlas for designing gB-based vaccines more closely mimicking the infectious virus.

## Teaser

Cryo-EM reveals two forms of a fusion protein on the surface of herpes simplex virus, informing vaccine design.

## INTRODUCTION

There are nine species of human herpesviruses that cause a wide range of diseases, from mild ailments to life-threatening cancers. They are classified into three subfamilies: α-, β-, and γ-herpesviruses (*1*). Herpes simplex virus type 1 (HSV-1), a member of the α-herpesvirus subfamily, causes painful oral herpes (“cold sores”) and genital herpes (*2*). As one of the most extensively studied viruses, HSV-1 also serves as a model system for advancing our understanding of the fundamental aspects of herpesvirus biology. All herpesvirus virions share a similar architecture, consisting of a lipid bilayer viral envelope surrounding an amorphous tegument layer and an icosahedral capsid that packages the viral genome. The surface of the viral envelope is adorned with over a dozen viral-encoded glycoproteins (*3*, *4*), which include essential proteins responsible for viral attachment and entry (*5*–*8*), as well as those with modulatory roles, such as immune evasion (*9*–*13*). Among them, glycoprotein B (gB) is particularly significant, as it mediates the fusion of the viral envelope with the host cell membrane, initiating infection (*14*). These glycoproteins are primary targets for herpesvirus vaccine development.

Like many other viral fusogens, gB exists in two distinct conformations: prefusion and postfusion. Prefusion gB is metastable and readily transitions to the postfusion conformation (*15*). Structure-based design of viral fusogens stabilized in their prefusion conformation holds great promise for improving their efficacy as vaccine. This approach has been successfully demonstrated in recent vaccine development efforts against respiratory syncytial virus (RSV) and severe acute respiratory syndrome coronavirus 2 (SARS-CoV-2) (*16*–*20*). Despite considerable efforts, an atomic structure of prefusion gB has remained elusive until recently, presenting a major hurdle to rational vaccine design. The recent success in determining the atomic structure of human cytomegalovirus (HCMV) gB in its prefusion form by cryo–electron microscopy (cryo-EM) marks a significant advance (*21*). In this case, the gB machinery was stabilized by a highly specific small-molecule fusion inhibitor and a nonspecific chemical cross-linker prior to detergent solubilization from the HCMV virion, followed by purification with a monoclonal antibody. Guided by the HCMV gB structure, stabilizing mutations have been designed and tested in the gB proteins of several other human herpesviruses, enabling visualization of their prefusion-like structures (*22*–*25*). These include two reports of HSV-1 gB atomic structures during the submission of this manuscript. Among these structures, HSV-1 gB displayed a marked divergence from other homologs, in which the central helix bundle that is tightly packed in others becomes splayed at the crest in HSV-1 gB. This structural divergence was also observed between previous low-resolution structures of membrane-anchored HCMV gB and HSV-1 gB visualized by cryo–electron tomography (cryo-ET) and subtomogram averaging (*26*, *27*). However, all these prefusion-like HSV-1 gB structures reported so far harbor a common point mutation, His^516^→Pro (H516P), which was believed to have stabilized the prefusion conformation (*26*). Whether the observed structural divergence reflects an authentic structural feature of HSV-1 gB or arises as a consequence of the point mutation remains to be clarified and is critical for structure-based vaccine design.

## RESULTS

### HSV-1 virion–derived gB displayed both prefusion and primed conformations

To visualize the native conformational landscape of gB, we optimized the procedure of solubilizing gB from HSV-1 virion with minimal manipulation for cryo-EM structural studies. First, we observed that during HSV-1 culture, extensive cell lysis often led to significant pH drop of the media, which may trigger prefusion to postfusion transition of gB. To mitigate that, we harvested the viral culture at an earlier time point than usual [48 versus 72 hours postinfection (hpi)] (*28*), sacrificing the production yield for the preservation of prefusion gB. Second, we skipped density gradient purification to avoid damage of viral surface proteins by the usually hypertonic gradient medium. Third, we performed tandem size exclusion chromatography (SEC) after detergent solubilization to effectively separate gB from most contaminants based on their size differences and to concentrate gB in the peak fractions (fig. S1A). This optimized workflow was proven to be effective in preserving the native prefusion structure of gB.

Despite the sharp peak observed in SEC, the sample appeared highly heterogeneous in cryo-EM imaging (fig. S1B). Initial two-dimensional (2D) classification only revealed class averages of a dominating contaminant later identified as α-2-macroglobulin (α2M) (fig. S1C), likely from the fetal bovine serum supplemented for cell culture (*29*). After excluding these α2M particles in a subsequent round of 2D classification, two class averages emerged, appearing to be the side view and top view of gB, respectively (fig. S1D). With these few thousand gB-like particles, we generated an ab initio 3D map that resembled the prefusion structure of HCMV gB. Using this map as an initial model, we fished out many more prefusion gB-like particles from the original dataset through 3D classification (fig. S1E). Notably, we were unable to retrieve any postfusion gB particles even when using a well-defined 3D structure as bait. Based on our experience, this was likely because the gB proteins were easily entangled during the prefusion to postfusion transition in a highly crowded mixture.

**Fig. 1.**
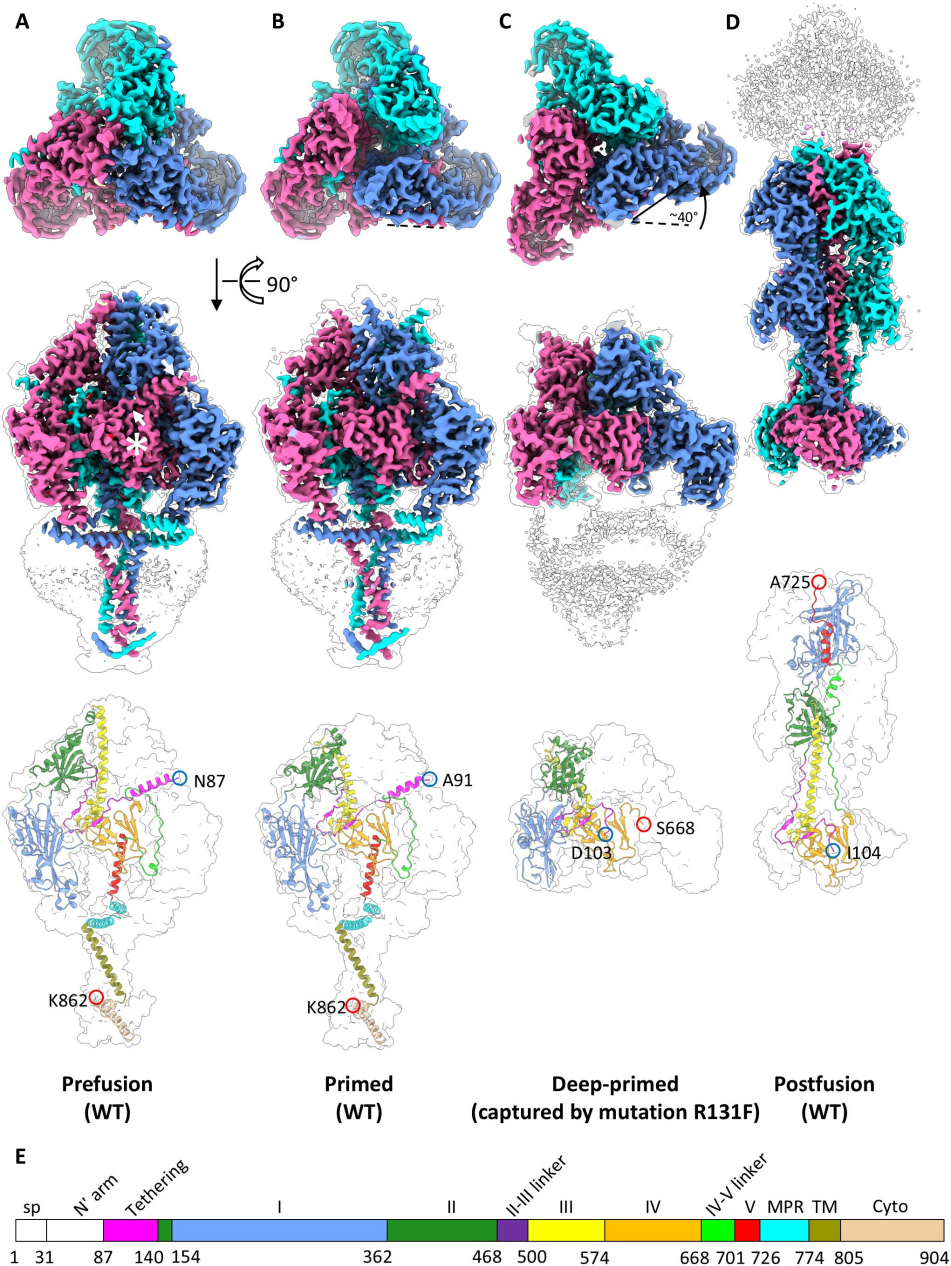
Cryo-EM structures of HSV-1 gB at different states along the conformation transition pathway. (**A**) The canonical prefusion state. (**B**) The primed state. (**C**) The deep-primed state captured by a mutant m3 (R131F). (**D**) The postfusion state. The three subunits in the gB trimer are colored differently with atomic models. The side views (middle row) of the four structures are roughly aligned by their domain IV densities [denoted by a white asterisk in the middle panel of (A)]. A white ghost of the density map shown at a very low threshold is overlapped with the structure. The top views (except for the postfusion state) are shown in the top row. Note the cracking in the primed state (B) and a roughly 40° counterclockwise rotation in the deep-primed state (C). White arrowheads in the middle panel of (A) point to densities that disappear at similar location in the primed state [middle panel of (B)]. In the bottom row, the atomic model of a single subunit is shown for each structure, with a white ghost of the entire trimer displayed in the background. The amino and carboxyl termini of the resolved region are denoted by blue and red circles, respectively, with the corresponding residues labeled. Domain coloring of the atomic model is according to that defined in (E). (**E**) Linear mapping of HSV-1 gB domains on its sequence. The boundary amino acid numbers are indicated at the bottom. sp, signal peptide; N′ arm, N-terminal arm (flexible and not resolved in the structure). The others are self-explanatory. This domain color scheme is maintained through all figures unless otherwise indicated.

Further 3D classification of the prefusion gB-like particles revealed two distinct structural populations (fig. S1E). The predominant population (73% of particles) exhibited a compact central helix bundle with a closed tip (Fig. 1A), similar to the locked prefusion conformation of HCMV gB (*21*). In contrast, the minor population (27%) displayed a cracked appearance when viewed from the membrane-distal end of the trimer (Fig. 1B), resembling the other reported prefusion-like structures of HSV-1 gB bearing the H516P mutation (*23*, *24*, *26*). We propose that the closed structure represents the authentic prefusion conformation of HSV-1 gB, whereas the slightly opened one represents an early intermediate, which we name the primed state, along the conformational transition pathway toward membrane fusion, even though we cannot rule out the possibility that this conformational difference simply reflects the intrinsic dynamics of gB.

HSV-1 gB has been proposed to assemble into a higher-order supercomplex with glycoprotein D (gD) and glycoproteins H and L (gHgL) that facilitates receptor binding–triggered activation of the gB fusogen (*30*). Despite extensive classification, we did not detect any gB subclass with extra density that could be attributed to a binding partner. Nonetheless, we cannot rule out the possibility that such supercomplexes are present on intact virions but were disrupted during detergent solubilization or with structural heterogeneity incompatible with single-particle averaging.

Consistently, later when we overexpressed and purified wild-type (WT) HSV-1 gB alone as a control for comparison with designed gB mutants, we also observed predominantly prefusion-like gB particles and only 10% postfusion particles (Fig. 1D and fig. S1, F to I). Although the exact proportions varied, a similar distribution of prefusion and primed states (82 and 8% of total gB particles, respectively) was observed, comparable to that in the virion-derived sample. These observations indicate that, at least in HSV-1, the gB fusion machinery does not require stabilization by a partner protein to maintain the prefusion conformation, and it likely exists in a dynamic equilibrium between the prefusion and primed states, rather than adopting a single static conformation.

Since the structures obtained from the overexpressed sample were indistinguishable from the virion-derived ones but reached higher resolution, we will use them for structural analyses below. The final resolution of these structures was 2.2 Å for the prefusion state, 2.8 Å for the primed state, and 2.5 Å for the postfusion state (fig. S2).

### Overall structure of the prefusion state of HSV-1 gB

**Fig. 2.**
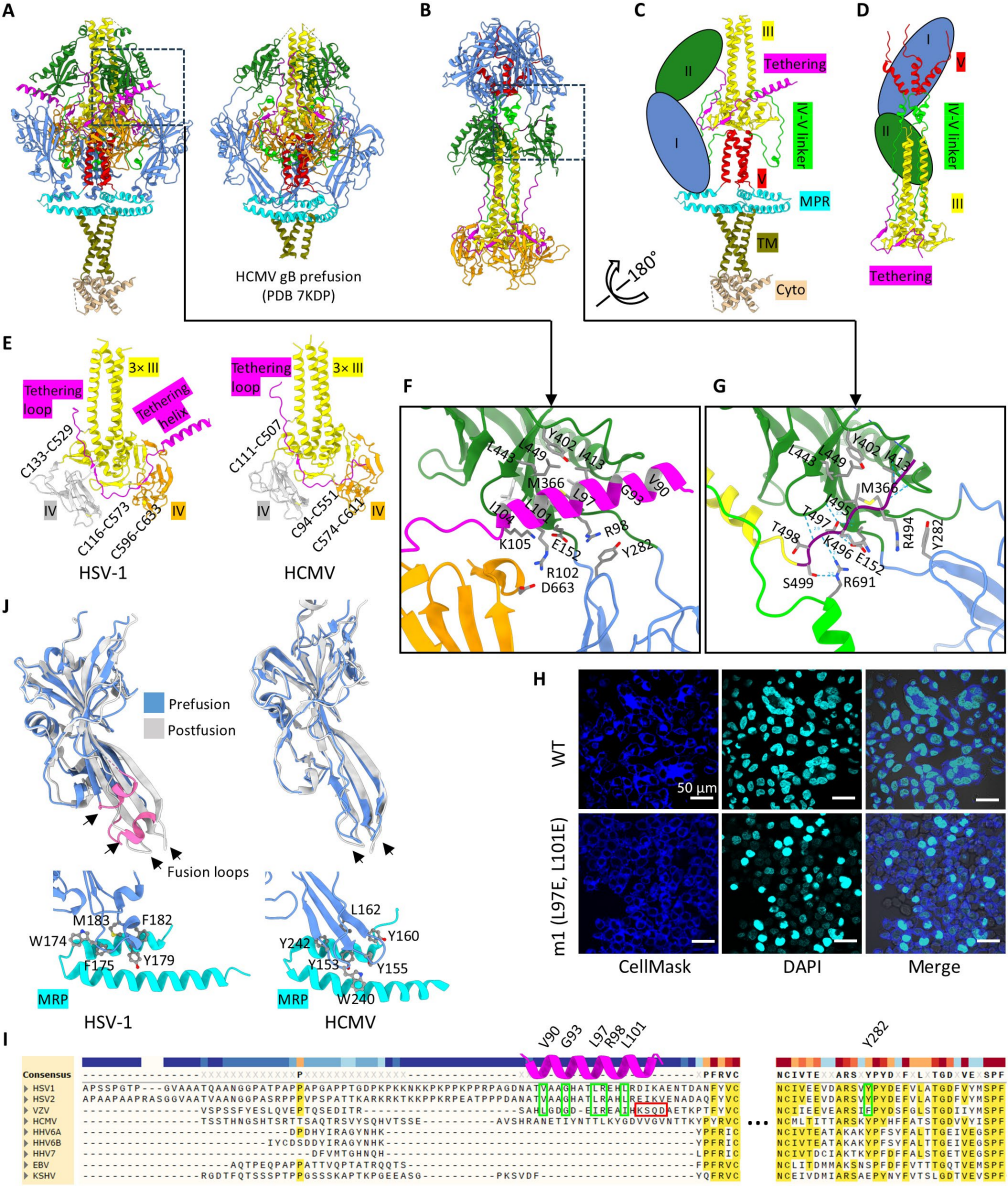
HSV-1 specific structural features. (**A** and **B**) Ribbon diagrams of HSV-1 gB prefusion (A) and postfusion states (B). The domains are color-coded as in Fig. 1E. A prefusion structure of HCMV gB (PDB 7KDP) is displayed for comparison. (**C** and **D**) Simplified representation of HSV-1 gB prefusion (C) and postfusion (D) structures with α-helix–rich central pillar (ribbons) and β-strand–rich periphery domains (ovals; only one copy is shown for simplicity). (**E**) Ribbon diagrams showing how the tethering domain is tethered to the central pillar via domain III and augmented by domain IV in both HSV-1 gB (left) and HCMV gB (right). Note the extra “tethering helix” in HSV-1 gB compared to HCMV gB. (**F**) Atomic interactions between the tethering helix and the domain I–domain II boundary in the prefusion gB. (**G**) Atomic interactions between a segment of the II-III linker (purple) and the domain I–domain II boundary in the postfusion gB. (**H**) Virus-free fusion assay comparing fusion activity between WT HSV-1 gB and gB mutant m1 (L97E, L101E). (**I**) Multiple sequence alignment of all human herpesvirus gB, highlighting the tethering helix and domain I–domain II boundary regions. The tethering helix of HSV-1 gB is marked on top. The green boxes highlight key residues mediating the tethering helix binding as shown in (F). Red box highlights the VZV gB sequence ^109^KSQD^112^ discussed in the text. (**J**) Comparing the fusion loop structures between prefusion gB and postfusion gB in both HSV-1 and HCMV. The fusion loops are pointed by black arrowheads. Key aromatic residues mediating the fusion loop and MPR interactions are highlighted in the bottom row.

Not unexpectedly, the prefusion structure of HSV-1 gB shares a similar morphology and domain architecture with those of HCMV gB (Figs. 1E and 2A) (*21*). Each gB machinery is a homotrimer with threefold rotational (c3) symmetry (Figs. 1 and 2, A and B). Following previous nomenclature (*14*, *21*), with necessary modifications, we define the domains of mature gB (residues 31 to 904, with the signal peptide, residues 1 to 30, removed) as follows (Fig. 1E and fig. S3). N-terminal arm, residues 31 to 86, is completely flexible and unresolved in our structure. Tethering domain, residues 87 to 139, is composed of a “tethering helix” and a long “tethering loop” (fig. S3B). Domain II, residues 140 to 153 and 362 to 467, consists of a rigid, eight-stranded β-barrel and three short helices (fig. S3C). Domain I, residues 154 to 361, is also a β-strand–rich domain that contains the fusion loops at its base (fig. S3D). II-III linker, residues 468 to 499, is located at the very tip of the gB trimer, entirely flexible and unresolved. Domain III, residues 500 to 573, features a characteristic “central helix” which, together with two other protomers, forms a coiled-coil helix bundle at the center of the trimer. Extending from the base of this bundle is a short helix and a β-hairpin that together create a rigid, petal-like platform for the attachment of other domains, such as domain IV and the tethering domain (Fig. 2, C and E, and fig. S3E). Domain IV, residues 574 to 667, forms a rigid β-sandwich stabilized by a highly conserved intradomain disulfide bond between Cys^596^ and Cys^633^ (fig. S3F); its structure and relative position to domain III remain largely unchanged between the prefusion and postfusion states, making it a reliable landmark for aligning gB structures in different conformational states (Fig. 1, A to D). The IV-V linker, residues 668 to 700, is a long coil folded into an ear-like shape with a short helix at the base resembling an earlobe (fig. S3G); in the prefusion structure, it is largely hidden beneath domain II and domain I; in contrast, in the postfusion structure, it becomes highly extended, with the coiled region inlaid into the surface groove of the central helix bundle of domain III (Fig. 2, A to D). Domain V, residues 701 to 725, comprises a single helix (fig. S3H) that assembles into a helix bundle with two other protomers. This helix bundle is positioned just beneath the central helix bundle of domain III (Fig. 2C), with the carboxyl group of Leu^545^ and the amide group of Tyr^702^ at their termini forming hydrogen bonds (not shown), resembling those found in a continuous α-helix. The membrane-proximal region (MPR), residues 726 to 773, forms an amphipathic α-helical hairpin that is partially embedded in the outer leaflet of the viral envelope membrane (fig. S3I and Fig. 1, A and B). The transmembrane (TM) domain, residues 774 to 804, marks the final portion of the structure that could be modeled ab initio (fig. S3J). The cytoplasmic (Cyto) domain, residues 805 to 904, appears mostly flexible, with only two helical densities resolved at subnanometer resolution (figs. S2A and S3K). However, the TM and Cyto domains can be fitted satisfactorily, as a rigid body, with the corresponding regions of the crystal structure of the postfusion gB [Protein Data Bank (PDB) 5V2S] (*15*), suggesting that they barely change from prefusion to postfusion. We keep this part in our atomic model for illustrative purposes.

The overall architecture of the prefusion gB can be simplified as an α-helix–rich central pillar and a β-strand–rich periphery. The central pillar comprises domain III, domain V, the MPR, the TM helices, and the Cyto domain from top to bottom, with the last three domains securing gB on the viral envelope like a rivet. The β-strand–rich domain II and domain I drape around the central pillar (Fig. 2, C and D), with the fusion loops at the bottom of domain I anchored on the surface of MPR (Fig. 2, A and C). This distinctive structural arrangement differentiates gB from other type III viral fusogens, such as the vesicular stomatitis virus glycoprotein (VSV-G) and baculovirus gp64, in which the fusion loops appear to be in direct contact with the viral envelope (*31*, *32*). A highly extended loop at the very N terminus of gB, which we define as the tethering domain, acts as an additional securing mechanism for domains II and I. This tethering loop is connected to a central β-strand that traverses domains II and I (fig. S3A). It tethers these two domains to the central pillar through β-strand addition interactions with domains III and IV in addition to two pairs of highly conserved disulfide bonds, Cys^133^–Cys^529^ and Cys^116^–Cys^573^ (Fig. 2, C to E). Despite the dramatic structural rearrangement of domains II and I during the transition from prefusion to postfusion, the tethering domain remains largely unchanged (Fig. 2, C and D). Notably, this tethering mechanism is conserved across all type III viral fusogens (Fig. 2E), including VSV-G and baculovirus gp64.

### HSV-1 specific structural features in the prefusion gB

Despite its similarity to HCMV gB and other type III viral fusogens, the prefusion structure of HSV-1 gB displays unique structural features with important functional implications. Notably, the tethering domain of HSV-1 gB features an additional helix at the very N terminus—here referred to as the “tethering helix”—which binds to a groove at the interface between domains I and II of an adjacent subunit, effectively cross-linking neighboring protomers (Fig. 2, A and F). The binding is primarily mediated by hydrophobic interactions between residues located on one side of this amphiphilic helix, including Val^90^, Gly^93^, Leu^97^, Leu^101^, and Ile^104^, and residues at the base of domain II. Additional significant interactions include the stacking of Arg^98^ with Tyr^282^ from domain I, charge-charge interactions between Lys^105^ and Glu^152^ from domain II, and the interaction of Arg^102^ with Asp^663^ from an adjacent domain IV (Fig. 2F). In the postfusion HSV-1 gB structure, this groove is occupied by a coil from the II-III linker (Fig. 2, B and G), which is entirely flexible in the prefusion gB structure. The interactions are somewhat similar to those described above, notably the hydrophobic interaction of Ile^495^ with the base of domain II and the stacking interaction between Arg^494^ and Tyr^282^ (Fig. 2G).

The tethering helix cross-links neighboring subunits, likely stabilizing the prefusion gB structure and coordinating the actions of its three subunits during the conformational transition. To assess the functional significance of this helix, we generated a gB mutant m1 with double mutation Leu^97^→Glu (L97E) and Leu^101^→Glu (L101E). The introduction of charged residues at the hydrophobic interface between the tethering helix and the domain II was expected to disrupt its binding. A virus-free fusion assay, in which human embryonic kidney (HEK) 293T cells coexpressing HSV-1 gB, gD, and gHgL were briefly treated with a low pH buffer to trigger gB action, showed that m1 completely lost its ability to induce cell-cell fusion, in stark contrast to the WT gB (Fig. 2H). Consistently, cryo-EM imaging of purified m1 revealed only the postfusion gB structure. These results highlight the critical role of the tethering helix in stabilizing the prefusion conformation of HSV-1 gB.

A multiple sequence alignment of gB from all nine human herpesvirus species reveals that the tethering helix is likely conserved in HSV-1, HSV-2, and varicella-zoster virus (VZV)—all belonging to the α-herpesvirus subfamily—but absent in the gB of β- and γ-herpesvirus subfamily members (Fig. 2I). A similar helix structure was observed in the recently reported HSV-1 and HSV-2 prefusion-like gB structures (*23*). A previous study had identified a sequence in this segment of VZV gB, ^109^KSQD^112^, corresponding to the last four residues of the tethering helix in HSV-1 gB (Fig. 2I), to be important for the fusion activity of VZV gB: Alanine substitution of these four residues abolished cell-cell fusion in a virus-free fusion assay and significantly impaired viral propagation when introduced into the viral genome (*33*). Our structural analysis now provides a possible mechanistic explanation for this observation: The four-alanine substitution may have somehow affected the binding affinity of the tethering helix, rendering the prefusion gB less stable and more prone to premature transitioning to the postfusion state.

Another distinctive feature in the prefusion gB of HSV-1 lies in its fusion loops. The fusion loops of all type III viral fusogens are positioned at the base of domain I [designated as domain IV in VSV-G (*31*)]. In all previously known structures, including both pre- and postfusion gB of HCMV (*21*, *34*) and the postfusion gB of HSV-1 (*14*), this region is organized into three β-strands and a coil. Membrane-inserting residues were believed to reside at or near the two tips of the strand-turn-strand and strand-turn-coil structures. In the prefusion gB of HCMV, both tips are in contact with the underlying MPR (*21*). In contrast, in the prefusion structure of HSV-1 gB, the two tips form short helices instead of strands. One helix interacts directly with the MPR, while the other is stacked above it (Fig. 2J). Similar structure was observed in the other HSV-1 prefusion-like gB reported recently (*23*). Unexpectedly, despite this significant structural difference, the molecular interactions between the fusion loops and the MPR are similar across HSV-1 and HCMV gB. In both cases, these interactions are primarily mediated by aromatic side chains from the fusion loop stacking against the exceptionally flat surface of the MPR helices from a neighboring subunit (Fig. 2J). In HCMV gB, these aromatic residues include Tyr^153^, Tyr^155^, Tyr^160^, Trp^240^, and Tyr^242^ from the two fusion loops, while in HSV-1 gB, they comprise Trp^174^, Phe^175^, Tyr^179^, and Phe^182^ from the lower helix. In addition, a hydrophobic residue—Leu^162^ in HCMV, corresponding to Met^183^ in HSV-1—also contributes to these interactions (Fig. 2J). Notably, a previous mutagenesis study based on the postfusion structure of HSV-1 gB had revealed that single mutations Trp^174^→Arg (W174R) or Tyr^179^→Lys (Y179K) nearly abolished the fusion activity of HSV-1 gB, while the Trp^174^→Tyr (W174Y) mutation retained approximately half of the WT activity (*35*). X-ray crystallographic structures of some of these gB mutants revealed no significant structural difference compared to the WT (*36*). The fusion-null phenotype of those mutations was interpreted as a result of charged residues disrupting the membrane insertion activity of the fusion loops. While this original explanation remains plausible, an alternative interpretation could be that the W174R and Y179K mutations may have significantly weakened the interactions between domain I and the MPR, leading to a less stable prefusion gB. Consistent with this interpretation, the aromatic side chain of tyrosine in the W174Y mutant allowed for a relatively strong interaction with the MPR, partially maintaining the stability of the prefusion conformation.

### Structural changes from prefusion to the primed state

**Fig. 3.**
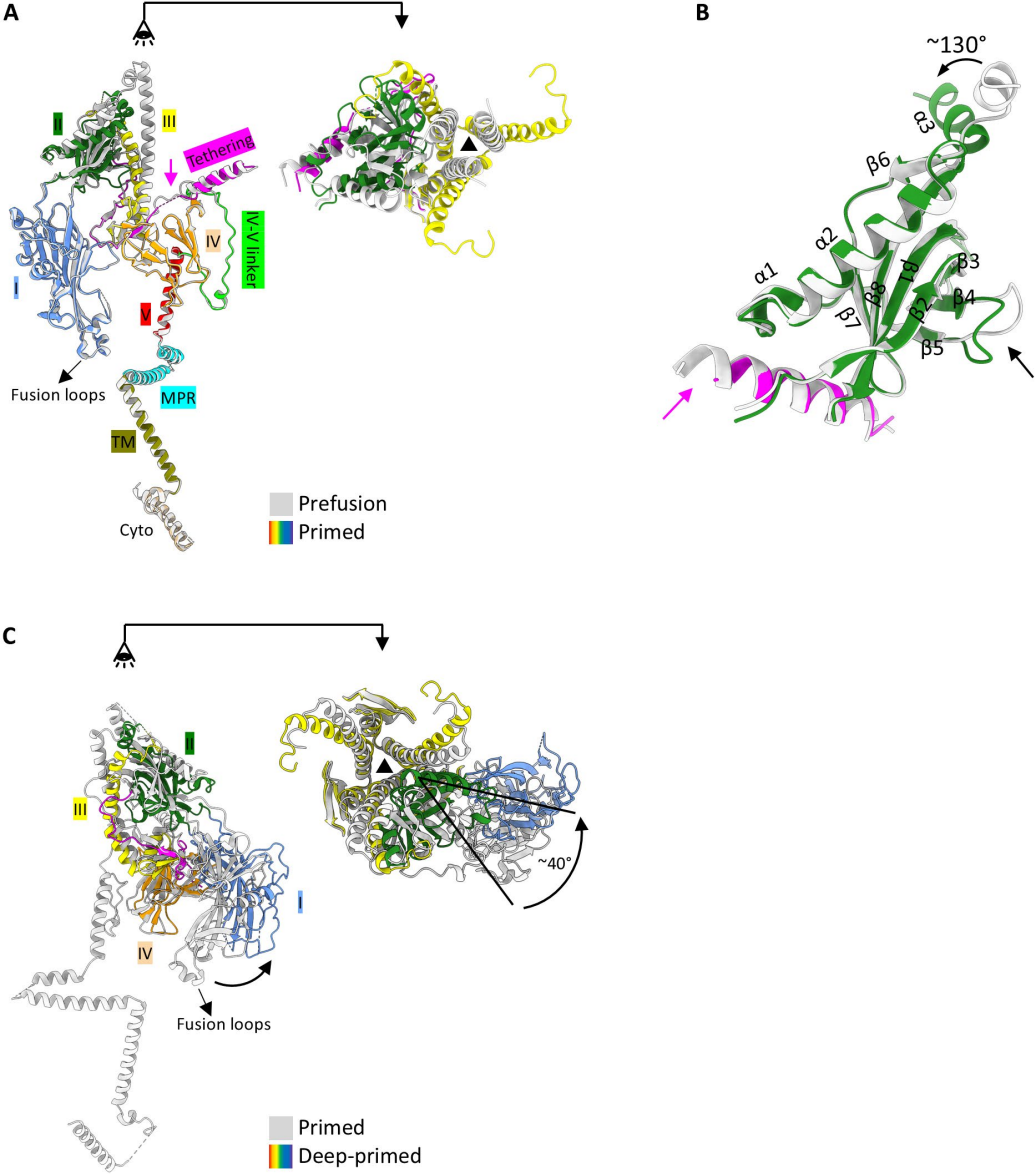
Structural changes that drive gB priming. (**A**) Superposition of gB models of the prefusion state (gray) and the primed state (colored). The magenta arrowhead points to a segment of the tethering loop that becomes flexible in the primed state. A top view in the right panel with the other two central helices displayed highlights the opening of the central helix bundle from prefusion to the primed state. The triangle indicates the c3 symmetry axis. (**B**) Superposition of domain II and the associated tethering helix models of the prefusion state (gray) and the primed state (colored) to identify structural change hotspots. Helix α3 in domain II undergoes a roughly 130° tilt. Black arrowhead points to structural change in a loop between β4 and β5. Magenta arrowhead points to the shortening of the tethering helix. (**C**) Superposition of gB models of the primed state and the deep-primed state. As in (A), a top view is displayed in the right to show the further opening of the central helix bundle. Domain II and domain I are tilted about 40° counterclockwise when measured by the orientation of the major helix in domain II (black lines), consistent with the measurement shown in Fig. 1C. Note that the consequence of this tilt would be the breakage of interactions between the domain I fusion loops and the MPR, as indicated by a curved arrow in the left panel.

**Fig. 4.**
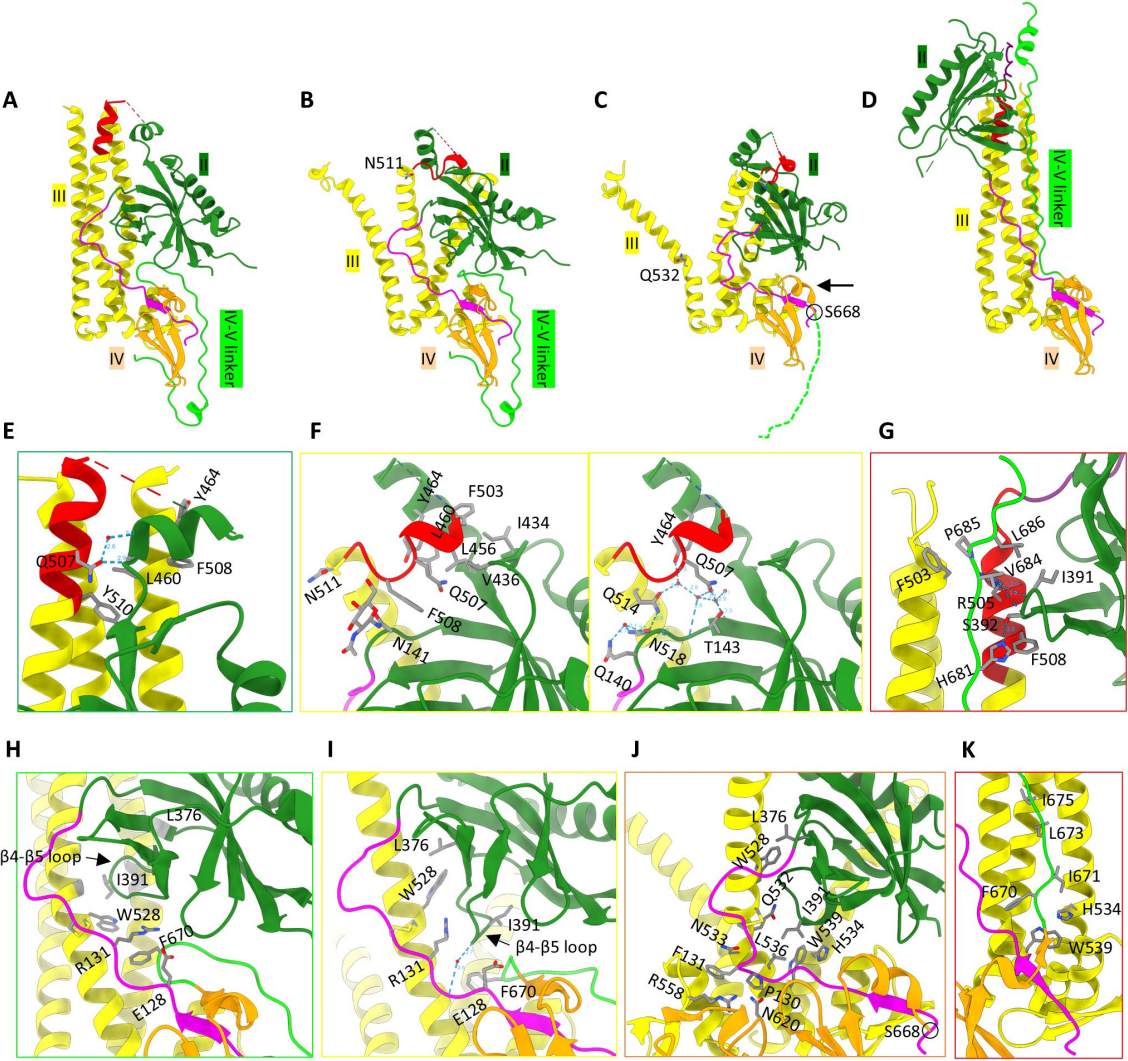
Key atomic interactions along the conformation transition pathway from prefusion to postfusion in HSV-1 gB. (**A** to **D**) Structural changes in the central helix bundle, domain II, and other key elements from the prefusion state (A), through the primed state (B), the deep-primed state (C), to the postfusion state (D). Domain coloring remains the same as defined in Fig. 1E except for one tip (highlighted in red) of the domain III central helices, which undergoes significant bending to engage with the domain II in the primed (B) and deep-primed (C) states. The kink positions of the central helix, Asn^511^ in the primed state (B) and Gln^532^ in the deep-primed state (C), are labeled. The dashed green line in (C) represents the flexible IV-V linker region. The circle marks the last residue resolved in the structure. Also note that the last segment of the domain IV refolds into a short helix (arrowhead) in the deep-primed state. (**E** to **G**) Atomic interactions between domain II and the tip of domain III central helix in the prefusion state (E), the primed state (F), and the postfusion state (G). Red dots are water molecules. Blue dashed lines represent hydrogen bonds. The two panels in (F) show the same structure but highlight different interactions separately for clarity. Structure of this region barely changes in the deep-primed state and thus is not shown. (**H** to **K**) Atomic interactions near the base of domain II in the prefusion state (H), the primed state (I), the deep-primed state (J), and the postfusion state (K).

Superposition of the gB model in the primed state with that of the prefusion state reveals three major structural changes: opening of the central helix bundle through slight bending of each central helix in domain III, partial unfolding and kinking at the tip of each central helix, and displacement of domain II (Figs. 3A and 4, A and B). Overall, these changes suggest that the three subunits, which form a closely packed trimer in the prefusion state (Fig. 1A), begin to dissociate in the primed state (Fig. 1B). However, this dissociation is limited as the three subunits remain cross-linked by the tethering helices (Fig. 1B). On the other hand, the tension exerted on the tethering helices by the dissociating trimer likely weakens their binding to the grooves, preparing them for complete dissociation in the next step. This is evidenced by the disappearance of densities corresponding to a helical turn at the N terminus and the loop at the C terminus of the tethering helices (magenta arrowhead in Fig. 3, B and A, respectively), both of which are well structured in the prefusion state (white arrowheads in Fig. 1A, middle).

Despite a notable displacement, the structure of domain II remains largely unchanged from the prefusion to the primed state. Superposition of domain II models from these two states reveals a highly rigid core, comprising an eight-stranded β-barrel and a “spine helix” (α2), along with two structural-change hotspots (Fig. 3B). The first hotspot is helix α3, spanning residues 459 to 467 at the C terminus of domain II, which undergoes a dramatic 130° tilt. The second hotspot is a short loop connecting β-strands β4 and β5 (hereafter referred to as the “β4-β5 loop”). In the prefusion state, these two regions serve as the main contact points between domain II and the central helix bundle of domain III (Fig. 4, A, E, and H). Helix α3 lies transversely along the side of the central helix bundle, with the side chains of Leu^460^ and Tyr^464^ buttressed by Tyr^510^ and Phe^508^ from the central helices, respectively. In addition, a water molecule mediates hydrogen-bond interactions between the side chain of Gln^507^ in domain III and the backbone amides of Leu^460^ and Ala^461^ in α3 (Fig. 4E). At the base, the side chain of Ile^391^ at the tip of the β4-β5 loop inserts into a hydrophobic pocket between two central helices, buttressed by Trp^528^ and surrounded by Leu^521^, Val^524^, and Ala^525^ (Fig. 4H).

The structural change in helix α3 of domain II is tightly coordinated with that of the central helix of domain III, which partially unfolds and kinks by ∼90° near residue Asn^511^ in the primed state (Fig. 4B). The side chain of Phe^503^ at the tip of the central helix now forms hydrophobic interactions with the side chains of Leu^460^ and Tyr^464^ from α3, as well as with Ile^434^, Val^436^, and Leu^456^ from the edge of the domain II β-barrel. In addition, the kinked segment of the central helix positions the side chains of Gln^507^ and Phe^508^ to stack against the side chain of Asn^141^ in domain II (Fig. 4F, left). The density corresponding to an N-linked glycan on Asn^141^, which is not observed in the prefusion structure, becomes well resolved in the primed state, suggesting stabilization of the Asn^141^ side chain through this stacking interaction. Furthermore, an intricate hydrogen-bond network, mediated by five water molecules, links polar side chains and backbone groups across domain II and the central helix of domain III (Fig. 4F, right).

At the base of domain II, the β4-β5 loop is released from the hydrophobic pocket above Trp^528^ as the central helix bundle splits. Domain II shifts downward by nearly two helical turns along the central helix. The side chain of Trp^528^ now points upward, buttressing domain II by propping against the side chain of Leu^376^ on β3 (Fig. 4I), which was ∼15 Å away in the prefusion structure (Fig. 4H). Collectively, these structural changes and newly established interactions transform domain II and domain III—from loosely associated in the prefusion structure (Fig. 4A)—into a tightly engaged, highly compact unit in the primed state (Fig. 4B).

**Fig. 5.**
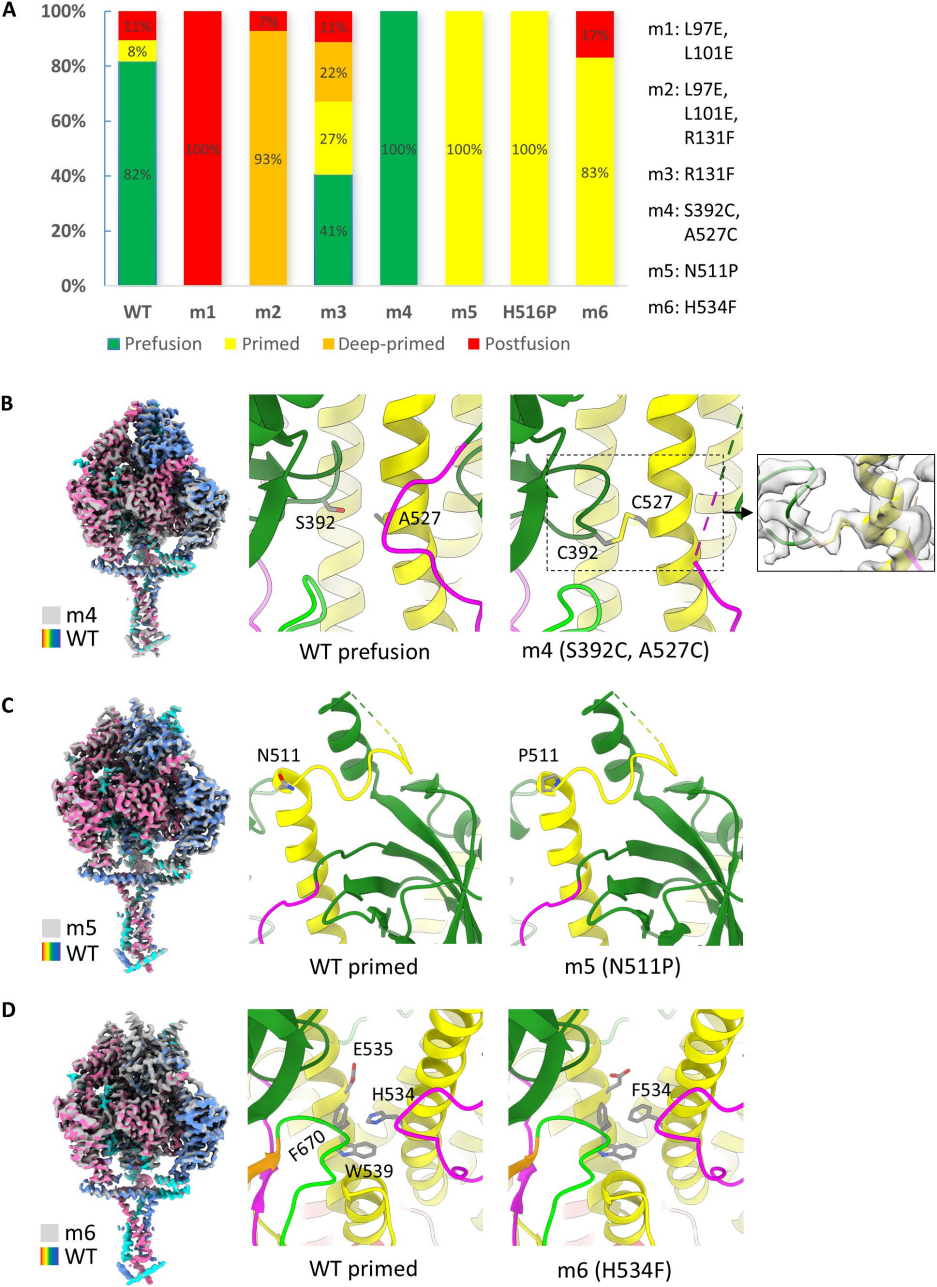
Structure-based design of gB mutants. (**A**) Conformational landscape of HSV-1 gB WT and mutants. Percentages of particles at different conformational states resolved by cryo-EM are plotted for the WT or mutants of HSV-1 gB. (**B** to **D**) Comparing structures of HSV-1 gB WT and stabilized mutant m4 (B), m5 (C), or m6 (D). In each row, the left panel shows superimposed density maps of gB WT and the dominating conformation of the stabilized mutant; the middle and the right panels compare the local structures between the WT (middle) and the mutant (right) around the mutational spot. The inset in the right panel of (B) highlights the electron density of the engineered disulfide bond.

The structural change and translocation of the β4-β5 loop appear to have significant functional implications, transmitting conformational changes from the tip of gB to its basal components, particularly the IV-V linker region. In the prefusion state, the IV-V linker is coupled to the tethering loop through a stacking interaction between the aromatic side chain of Phe^670^ and the side chain of Arg^131^ on the tethering loop, with Arg^131^ further stabilized by Glu^128^ (Fig. 4H). In the primed state, the β4-β5 loop inserts between Arg^131^ and Glu^128^, with a water molecule mediating hydrogen bonds between the carboxyl group of Ser^392^ on the β4-β5 loop and the carboxyl group of Gln^129^ on the tethering loop (Fig. 4I). This insertion disrupts the interactions among Arg^131^, Glu^128^, and Phe^670^, effectively decoupling the IV-V linker from the tethering loop. The Phe^670^ side chain is subsequently captured by nearby Trp^539^ and His^534^ on the central helices, forming a triangular stacking interaction (Fig. 5D, center). Notably, Phe^670^ marks the turning point of the IV-V linker during its transition from the prefusion to postfusion state. From this point, the IV-V linker swings nearly 180°, reorienting from a downward to an upward position (Fig. 4A versus Fig. 4D) and inserting a series of hydrophobic side chains (Ile^671^, Leu^673^, Ile^675^, Leu^678^, and Phe^683^) into the groove formed between two neighboring central helices (Fig. 4, G and K). The first of these, Ile^671^, is positioned directly above His^534^ in the postfusion gB structure (Fig. 4K). Thus, the conformational transition from the prefusion to the primed state brings the IV-V linker into proximity with the central helix bundle, priming it for the next step in the fusion process.

### Capturing the deep-primed state with a gB mutant

The disordering of densities around the tethering helix in the primed-state structure suggests that the tethering helices are on the verge of fully dissociating from their binding sites to liberate the cross-linked trimer for further conformational changes. Since the m1 mutant (L97E and L101E) has likely lost tethering helix binding, we reasoned that any additional stabilizing mutation on the m1 background might help capture a downstream intermediate of gB beyond the primed state. Out of nearly a dozen triple mutants (L97E, L101E, and a third point mutation), we identified one that could be purified and showed prefusion-like particles under cryo-EM. Three-dimensional classification and reconstruction of this mutant—L97E, L101E, and Arg^131^→Phe (R131F), hereafter referred to as m2—revealed a predominant (93% of particles; Fig. 5A) structure that closely resembled the primed state in terms of opened central helices. As expected, this structure lacked tethering helix densities at the domain I–domain II boundary (Fig. 1C). We designated this conformation the “deep-primed state” based on the following analysis.

A replicate single-point mutation on the WT background—R131F, hereafter referred to as m3—recapitulated the full spectrum of prefusion (41% of particles), primed (27%), deep-primed (22%), and postfusion (11%) structures (Fig. 5A). As expected, the tethering helix remained associated in the prefusion and primed states but was absent in the deep-primed and postfusion states. The prefusion and primed structures of m3 were nearly indistinguishable from their WT counterparts (fig. S4, A, B, D, and E), except that a coil at the beginning of the IV-V linker (residues 669 to 677) folded into a short helix (fig. S4, C and F). This structural change likely occurred because the mutated Phe^131^ residue failed to form π-π stacking with Phe^670^ on the IV-V linker to hold it in place, as Arg^131^ does in the WT structure (Fig. 4H and fig. S4C). This outcome was entirely unexpected, as our intention was to strengthen the stacking interaction by introducing an additional phenylalanine side chain. Nonetheless, the presence of prefusion and primed states in the m3 dataset confirmed that the deep-primed structure we captured represents a bona fide intermediate trapped along the prefusion to postfusion transition pathway.

### Structural changes from the primed state to the deep-primed state

The deep-primed structures from the m2 and m3 datasets were essentially identical and well resolved only in the upper regions of the extracellular domain. The last residue we could trace was Ser^668^, corresponding to the C terminus of domain IV. Regions beyond this point, including the IV-V linker, domain V, MPR, TM, and Cyto domains, are flexible and not resolved in our structure (Fig. 1C). Superimposition of the atomic model of the deep-primed state with that of the primed state revealed further separation of the central helices (Fig. 3C), as expected upon release of the constraints imposed by the tethering helices. Bending of the central helix is less uniform than in the primed state, with a notable kink observed around Gln^532^ (Fig. 4B versus Fig. 4C). The tip of each central helix remains partially unfolded and kinked, engaging domain II through the same atomic interactions observed in the primed-state structure (Fig. 4F). Most significantly, the translocation of domain II along with the central helix results in a nearly 40° counterclockwise (when viewed from the membrane-distal end of gB) rotation of domain II and domain I (Figs. 1C and 3C, right). In stark contrast to the primed state, where domain I remains stationary (Fig. 3A), this dramatic rotation effectively wrenches domain I off the MPR, explaining the relative flexibility of the unresolved IV-V linker, domain V, MPR, TM, and Cyto domains in the deep-primed structure. Consequently, conformational change from the prefusion/primed state to the deep-primed state unleashes the fusion loops from the tight engagement with MPR, readying them to flip over to the membrane-distal side of gB and insert into the target membrane.

In addition to the rotational movement, domain II also undergoes a slight downward shift (Fig. 3C), which brings the β4-β5 loop into proximity with the base of domain III. The side chain of Ile^391^ on this loop now forms hydrophobic and stacking interactions with Leu^536^, Gln^532^, Trp^539^, and His^534^ on the central helices of domain III (Fig. 4J). As in the primed state, the side chain of Trp^528^ on the central helix continues to support domain II by propping against the side chain of Leu^376^ (Fig. 4J). Because the tethering loop is covalently linked to the central helix via a disulfide bond between Cys^133^ and Cys^529^, the kink in the central helix around Gln^532^ appears to compress the lower portion of the tethering loop. This deformation results in a slight curving of the tethering loop backbone and flipping of the Phe^131^ side chain (corresponding to Arg^131^ in the WT) to the opposite side of the loop. The side chain of Phe^131^ now stacks between the side chains of Asn^533^ and Arg^558^ from domain III. In addition, the adjacent Pro^130^ forms stacking interactions with Leu^536^ from domain III and Asn^620^ from domain IV. The strong stacking interactions mediated by Phe^131^ likely explain how the R131F mutation stabilizes the transient deep-primed state for cryo-EM visualization. We speculate that in the WT structure, Arg^131^ could adopt a similar configuration, as its guanidinium group is capable of forming hydrogen bonds with several nearby polar side chains and structured water molecules in addition to stacking interactions. However, electrostatic repulsion between the positively charged side chains of Arg^131^ and Arg^558^—especially under acidic conditions—would disfavor this conformation and instead promote reversal of the structural changes described above, facilitating straightening and rebundling of the central helices.

### Structure-based design of HSV-1 gB mutants stabilized in the prefusion or primed state

A detailed understanding of the HSV-1 gB conformational changes informed our design of gB mutants stabilized in the prefusion or primed states for future vaccine evaluation. As the translocation of domain II—particularly its β4-β5 loop—appears to relay structural changes in the central helix bundle to other regions of gB, we speculated that constraining the relative position of the β4-β5 loop with respect to the central helix bundle could prevent spontaneous transition from the prefusion to the primed state. To test this, we introduced cysteine substitutions at Ser^392^ in the β4-β5 loop and Ala^527^ in the central helix (S392C, A527C), enabling disulfide bond formation between the two sites. Cryo-EM analysis of this mutant, designated m4, revealed exclusively prefusion particles, with no detectable primed or postfusion conformations (Fig. 5A), validating the effectiveness of this stabilization strategy. Structural comparison with the WT confirmed that m4 adopts a bona fide prefusion conformation (Fig. 5B, left), despite local structural deviations in the β4-β5 loop (Fig. 5B, middle and right).

To stabilize gB in the primed state, we introduced an Asn^511^→Pro (N511P) mutation at the site where the central helix of domain III kinks in transition to the primed and then the deep-primed state (Fig. 4, B and F). Because proline is generally disfavored in α-helices, we anticipated that this substitution would destabilize the straight central helix in the prefusion state, thereby facilitating the transition to the primed state. Moreover, this proline would stall the refolding of the kinked helix back to the straight configuration, thereby preventing progression to the postfusion state and effectively locking gB in the primed state. Consistent with this hypothesis, cryo-EM analysis of the resulting mutant, designated m5, revealed 100% primed particles (Fig. 5A). As expected, the structure of m5 was highly similar to the WT primed state, with a root mean square deviation (RMSD) of 0.258 Å (Fig. 5C).

The previously designed stabilizing mutation in HSV-1 gB, H516P, is located approximately one and a half helical turns downstream of Asn^511^ along the central helix. The subnanometer-resolution structure of this mutant closely resembled the WT primed state observed in this study (*26*). To gain a deeper understanding of its stabilizing mechanism and compare it with the N511P mutation, we determined the atomic structure of this H516P mutant by single-particle cryo-EM. As expected, and consistent with the previous cryo-ET and subtomogram averaging analysis (*26*), all particles adopted a conformation resembling the primed state (Fig. 5A and fig. S5A). In this structure, the central helix of domain III still kinked at Asn^511^, while the Pro^516^ residue retained an α-helical conformation (fig. S5C). This observation reinforces that the primed state represents a well-defined conformation and that the stacking interactions mediating the tight engagement between domain II and domain III are highly specific. This specificity likely reflects their functional importance in priming gB. We speculate that the primed state–stabilizing effect of H516P mutation acts through a mechanism similar to that of N511P—by disfavoring the straight configuration of the central helix in both the prefusion and postfusion states.

Although domain II and domain III are the primary drivers of the conformational transition, targeting other translocated structural elements—such as the IV-V linker—offers an alternative strategy for stabilizing intermediate states. As described above, Phe^670^ on the IV-V linker forms a stacking interaction with Arg^131^ on the tethering loop in the prefusion conformation (Fig. 4H); upon transitioning to the primed state, Phe^670^ is released from Arg^131^ (Fig. 4I) and subsequently captured by His^534^ and Trp^539^ on the central helices, forming a unique triangular stacking interaction (Fig. 5D, center). To mimic and potentially reinforce this triple stacking, we substituted His^534^ with phenylalanine (H534F, designated m6). Unexpectedly, this mutation appeared to have destabilized the prefusion conformation, as no prefusion particles were observed in the cryo-EM dataset (Fig. 5A). Nonetheless, the predominant conformation adopted by the H534F mutant was highly similar to the WT primed state with an RMSD of 0.805 Å (Fig. 5D, left), demonstrating its effectiveness in stabilizing the primed state as we expected. In retrospect, we speculate that the destabilization of the prefusion state may result from steric hindrance and/or incompatibility between the bulky, hydrophobic phenylalanine side chain and the adjacent charged residue Glu^535^ on a neighboring central helix (Fig. 5D), which are close to each other in the prefusion state. Consistent with the N511P and H516P mutations, this observation further demonstrates that destabilizing the central helix bundle in domain III will drive spontaneous transition of the prefusion state toward the primed state.

## DISCUSSION

Here, we present the least perturbed conformational landscape of HSV-1 gB among all herpesvirus fusogens characterized to date and demonstrate that it exists in an equilibrium between the dominant prefusion state and a minor population of primed intermediate. This divergence from the previously characterized HCMV gB likely arises from the presence of an additional tethering helix at the N terminus of HSV-1 gB, which cross-links the prefusion trimer, reinforces structural integrity, and imposes a kinetic barrier along the conformational transition pathway. Sequence analysis suggests that a similarly stable primed state may also exist in other α-herpesviruses, such as HSV-2 and VZV, but not in β- or γ-herpesviruses. These findings have potential implications for herpesvirus vaccine design. For α-herpesviruses, both prefusion-stabilized and primed state–stabilized gB mutants, or one that can adopt both conformations, may better mimic the native conformational landscape of gB on the infectious virion compared to a single-conformation construct. In contrast, for β- and γ-herpesviruses, the primed state is predicted to be highly transient, suggesting that vaccine strategies can focus solely on the prefusion conformation.

Structural comparison of prefusion, primed, and deep-primed gB reveals a priming mechanism in which structural changes in the central helices disrupt two key stabilizing interactions that maintain the prefusion conformation: cross-linking by the tethering helices and the association between domain I and the MPR. Although a stable primed state has not been observed in HCMV gB (*21*) or in other stabilized prefusion-like gB structures reported in two recent preprints (*22*, *25*)—likely due to the absence of the tethering helix in β- and γ-herpesviruses (Fig. 2I)—we propose that the priming mechanism is conserved across all human herpesvirus gB homologs. Supporting this hypothesis, multiple sequence alignment of all human herpesvirus gB proteins reveals conserved residues corresponding to Asn^141^, Gln^507^, and Phe^508^ (fig. S6)—key residues that mediate stacking interactions between domain II and domain III during priming in HSV-1 gB (Fig. 4F, left).

**Fig. 6.**
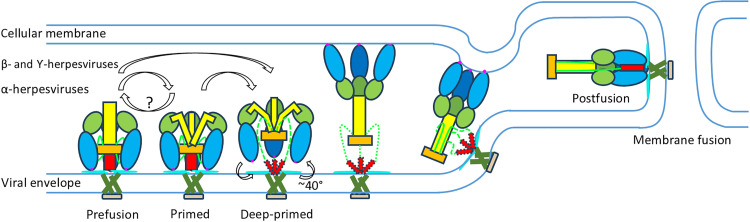
Schematic representation of herpesvirus gB-mediated membrane fusion. The two intermediate steps that are not labeled are imaginary. The tethering domain and α-herpesviruses–specific cross-linking by the N-terminal tethering helix are omitted for clarity. We propose that in β- and γ-herpesviruses, a stable primed state does not exist owing to the lack of the N-terminal tethering helix; instead, the prefusion gB shifts directly to the deep-primed state. The question mark indicates that it is not clear whether the primed state can shift back to the prefusion state in α-herpesviruses.

Beyond the deep-primed state, we propose the following downstream steps in the gB conformational transition pathway (Fig. 6), which are consistent with a previously proposed general scheme for viral membrane fusion (*37*). First, the bent and unbundled central helices of domain III observed in the primed and deep-primed states would straighten and rebundle, as seen again in the postfusion structure (Fig. 2, B and D). Concurrently, domains II and I would flip to insert the fusion loops at the tip of domain I into the target membrane. Subsequently, the freed IV-V linkers would thread into surface grooves along the central helix bundle of domain III, beginning at its base. This threading would reorient the gB ectodomain from a membrane-perpendicular to a more membrane-parallel configuration, thereby drawing the target membrane—engaged by the fusion loops—into close apposition with the viral envelope, which remains anchored via the MPR, TM, and Cyto domains, to drive membrane fusion (Fig. 6). Among these steps, the flipping of domains II and I represents the most dramatic structural rearrangement and likely constitutes the major energetic barrier. We propose that this step is coupled to—and driven by—the rebundling of the central helices in domain III. As described above, domain II is only loosely associated with the central helix bundle in the prefusion state but becomes tightly engaged with the kinked tip of the helices in the primed and deep-primed states. Upon straightening and rebundling of these kinked central helices, the tightly associated domain II—and the pendant domain I—would be hurled toward the opposite end of the helix bundle. In this sense, the bending of the central helices during priming serves as a spring-loading mechanism that stores the energy required to launch the fusion loops into the target membrane.

During the submission of this manuscript, Vollmer *et al.* (*23*) published atomic structures of HSV-1 gB stabilized with four point mutations—H516P, Ser^392^→Cys (S392C), Gln^532^→Cys (Q532C), and Asn^709^→Val (N709V)—with and without the binding of a neutralizing nanobody Nb1_gbHSV. Later, Roark *et al.* (*24*) also published several atomic structures of HSV-1 gB ectodomain stabilized by various mutations, with H516P included in all of them, and characterized a “prefusion-specific” gB-neutralizing antibody WS.HSV-1.24. It is worth clarifying that both so-claimed prefusion structures of HSV-1 gB actually represent the minor-populated primed state of HSV-1 gB on the virion surface. As the epitope of Nb1_gbHSV is located across domain I and domain IV, and these two domains barely change from the prefusion state to the primed state in our structures (Fig. 3A), it is expectable that Nb1_gbHSV can bind to the prefusion state of HSV-1 gB as well. This explains why this nanobody raised against the primed state of HSV-1 gB can still neutralize HSV-1 and HSV-2 infections at high efficacy even though the prefusion state is expected to dominate on the virion surface. Similarly, the epitope of WS.HSV-1.24, which is at the domain I–domain II boundary, barely has any structural change from prefusion to the primed state, even though domain II itself is notably translocated (Fig. 3A).

A hypervariable region (HVR) spanning ∼15 to 55 residues is present in all gB sequences, corresponding to residues 468 to 499 in HSV-1 gB (fig. S7A). A furin cleavage site had been identified within this region for some gB (*38*, *39*), particularly in β- and γ-herpesviruses (fig. S7A), but their functional significance remains debated (*40*, *41*). Structurally, the HVR connects domain II to the central helix of domain III (fig. S7B)—therefore referred to as the II-III linker in our structural description—but due to its high flexibility, it remains completely unresolved in both our prefusion and primed structures. A short segment at the C terminus of this region (residues Val^492^ to Ser^499^) becomes well resolved in the postfusion structure of HSV-1 gB [Fig. 2G and (*14*)], where it binds a groove on domain II that, in the prefusion conformation, is occupied by the tethering helix (Fig. 2F). Structurally, the presence of a long, flexible sequence in this region appears unnecessary, as the resolved C terminus of domain II and the N terminus of domain III are separated by only a few angstroms in both the prefusion and primed structures (Fig. 4, A and B). We propose that the HVR may function as a conserved immune evasion mechanism: The presence of three highly flexible HVR copies at the apex of the prefusion trimer masks an otherwise protruding and likely highly immunogenic epitope (fig. S7B). Furin cleavage in the middle of this region may further enhance its flexibility and, in turn, its capability to evade immune surveillance. This apex represents the most significant, if not the only, epitope that is present in the prefusion structure but absent in the postfusion structure. Masking this epitope by the HVR might be one of the reasons why stabilized prefusion gB of HCMV did not perform better than its postfusion form in terms of neutralizing antibody titers and protection against viral infection as recently reported (*42*, *43*). Whether removing or engineering this HVR will enhance immune recognition and vaccine potency deserves further testing.

## MATERIALS AND METHODS

### Cell culture

Vero cells [American Type Culture Collection (ATCC) CCL-81] and HEK293T/17 cells (ATCC CRL-11268) were cultured in Dulbecco’s modified Eagle’s medium (Sigma-Aldrich) supplemented with 10% fetal bovine serum (Corning) at 37°C with 5% CO_2_. Sf9 insect cells (Thermo Fisher Scientific) were cultured in HyClone CCM3 serum-free medium (Cytiva) at 27°C. Expi293F suspension cells (Gibco) were cultured in Expi293 expression medium (Gibco) at 37°C with 6% CO_2_ and 80% humidity. The cell lines were routinely checked to be negative for mycoplasma contamination but have not been authenticated.

### HSV-1 virion–derived gB purification

Preparation of HSV-1 virion followed previously described protocols (*28*, *44*). Briefly, Vero cells were cultured to 90% confluence and inoculated with HSV-1 (strain KOS, ATCC VR-1493) at a multiplicity of infection (MOI) of ∼0.01. At 48 hpi, the culture medium was collected and centrifuged at 10,000*g* for 20 min to remove cell debris. Viral particles were pelleted by ultracentrifugation at 100,000*g* for 2 hours. The pellet was incubated in NT buffer (150 mM NaCl and 50 mM tris-HCl, pH 8.0) overnight on ice and then gently resuspended. Usually, ∼5 ml of virus suspension was collected for each batch of purification. To solubilize the viral glycoproteins, a 10% DDM/CHS premade solution [dodecyl-β-d-maltoside (100 mg/ml) and cholesteryl hemisuccinate-tris (10 mg/ml), Anatrace] was added to the virus suspension at 1:10 ratio and incubated at 4°C for 2 hours with gentle shaking. The mixture was centrifuged at 10,000*g* for 20 min to remove aggregated viral capsids, tegument proteins, and other junk. The clarified virus soup was concentrated down to 500 μl with a 100-kDa molecular weight cutoff (MWCO) Amicon ultrafiltration device (Millipore). The mixture was then separated by SEC in a Superdex 200 Increase 10/300 GL column (Cytiva) pre-equilibrated in a sample buffer of 150 mM NaCl, 50 mM tris-HCl (pH 8.0), and 0.02% DDM/CHS.

### Expression and purification of WT or mutant gB

The DNA sequence expressing full-length mature gB (amino acids 31 to 904), with an influenza hemagglutinin signal peptide and a 3×FLAG tag added at the amino terminus, was synthesized and cloned into pBMCL1 vector at GenScript. pBMCL1 was a gift from L. Chen (Addgene plasmid no. 178203; http://n2t.net/addgene:178203; RRID: Addgene_178203) (*45*). All gB mutants were generated from this construct with the Q5 site-directed mutagenesis kit (New England Biolabs) and verified with plasmid sequencing (Plasmidsaurus or Azenta). Baculoviruses were generated from these constructs following the standard procedure described for the Bac-to-Bac system (Thermo Fisher Scientific). For protein expression, the passage 2 (p2) baculovirus stock was added to Expi293F cells at a density of 3 × 10^6^ cells/ml in 1:10 volume ratio. At 12 hpi, sodium butyrate was added to the culture at a final concentration of 10 mM to boost protein expression. After another 48 hours, cells were harvested by centrifuging at 5000*g* for 15 min at 4°C. For protein purification, the pellet from each 500 ml of cell culture was resuspended in 40 ml of lysis buffer [150 mM NaCl, 50 mM tris-HCl (pH 8.0), and Roche cOmplete protease inhibitor cocktail] and then lysed by sonication. After centrifugation at 4000*g* for 10 min to remove large debris, the lysate was mixed with 10% DDM/CHS to a final concentration of 1% and incubated for 2 hours at 4°C with gentle shaking. The insoluble fraction was then removed by centrifugation at 50,000*g* for 30 min. A 500-μl slush of anti-FLAG M2 resin (Sigmal-Aldrich) was added to the solution and incubated for 1 hour. After gravity flow through a chromatography column (Bio-Rad), the resin was washed twice with 10 ml of washing buffer [150 mM NaCl, 50 mM tris-HCl (pH 8.0), and 0.05% DDM/CHS]. The target protein was then eluted with 3 ml of elution buffer [150 mM NaCl, 50 mM tris-HCl (pH 8.0), 0.05% DDM/CHS, and 3×FLAG peptide (200 μg/ml)], concentrated down to 500 μl, and purified by SEC in the same way as described above.

### Cryo-EM sample preparation, data collection, and data processing

For cryo-EM sample preparation, the SEC peak fractions of purified WT or mutant gB proteins were concentrated to ∼1 mg/ml or the minimum volume achievable (∼30 μl) with a 100-kDa MWCO Amicon ultrafiltration unit. An aliquot of 3.5-μl sample was applied to a glow-discharged 300-mesh Quantifoil R1.2/1.3 Cu grid, blotted with filter paper, and plunge-frozen in liquid ethane with Vitrobot Mark IV (Thermo Fisher Scientific). Cryo-EM data collection was done with serialEM on a Titan Krios microscope equipped with Gatan BioQuantum K3 imaging filter and camera. A 20-eV slit was used for the filter. Images were recorded at 130,000× magnification, corresponding to a pixel size of 0.33 Å/pixel at super-resolution mode of the camera. A defocus range of −1.0 to −1.8 μm was set. A total dose of 50e^−^/Å^2^ at dose rate of ∼13e^−^/pixel/s was fractionated into 50 frames. The first two frames of each movie stack were excluded in motion correction. A binning factor of 2 was applied during motion correction, leading to a pixel size of 0.66 Å/pixel for the micrographs. Cryo-EM data processing was done with cryoSPARC (*46*), following procedures as detailed in fig. S1.

### Model building and refinement

Initial atomic model of the prefusion gB was built manually in Coot with individual domains of the postfusion gB structure (PDB 2GUM) (*14*) fitted with ChimeraX (*47*) and used as reference to help trace the backbones. The model was refined with Phenix real-space refinement program (*48*) and manually checked again in Coot (*49*). This process was iterated for several cycles until no significant improvement of the model was observed. Models of the gB mutants were modified from the WT and polished in the same procedure. Structure display and figure preparation were done with ChimeraX (*47*).

### Fusion assay and fluorescence microscopy

DNA sequences expressing HSV-1 gH, gL, and gD were synthesized and cloned into pBMCL1 individually at GenScript. The expression cassettes of gH and gL were merged into one vector with the LINK sequences (*45*, *50*). Baculoviruses expressing gHgL or gD were generated in the same way as those expressing gB (WT or mutant) as described above. For gB-mediated cell fusion assay, HEK293T cells were grown to a monolayer on coverslips in a six-well plate, and inoculated with p2 baculoviruses expressing gHgL, gD, and WT/mutant gB at MOI = 5 for each virus. At 48 hpi, the cells were washed three times with pH 7.4 phosphate-buffered saline (PBS; Gibco), twice with physiological saline solution (0.85% NaCl), and then treated with citric acid–sodium citrate buffer (0.1 M, pH 4.5) for 3 min at room temperature. After the treatment, cells were washed three times with PBS (pH 7.4), added with culture medium, and incubated at 37°C for 4 hours. Then, the cells were washed three times with PBS (pH 7.4) and fixed by 4% paraformaldehyde for 10 min at room temperature. Last, the cells were washed three times with PBS (pH 7.4), stained by CellMask Plasma Membrane Stain (Invitrogen), and mounted with ProLong Diamond Antifade Mountant with 4′,6-diamidino-2-phenylindole (DAPI; Invitrogen). Fluorescence images were captured using an Olympus FluoView FV3000 confocal laser scanning microscope and processed with ImageJ.
