# Supplementary material for: The native conformational landscape and priming mechanism of herpes simplex virus glycoprotein B
Source: Sci Adv. 2026 Jul 24;12(30):eaed8023. doi: 10.1126/sciadv.aed8023 (PMC13398477; doi:10.1126/sciadv.aed8023)
Supplement: Supplementary file 1 — Figs. S1 to S7 Table S1 [file sciadv.aed8023_sm.pdf]

Science Advances
AAAS

Supplementary Materials for

The native conformational landscape and priming mechanism of herpes simplex virus glycoprotein B

Zongjun Mou *et al.*

Corresponding author: Xinghong Dai, xinghong.dai@case.edu

Sci. Adv. **12**, eaed8023 (2026)
DOI: 10.1126/sciadv.aed8023

This PDF file includes:

Figs. S1 to S7
Table S1

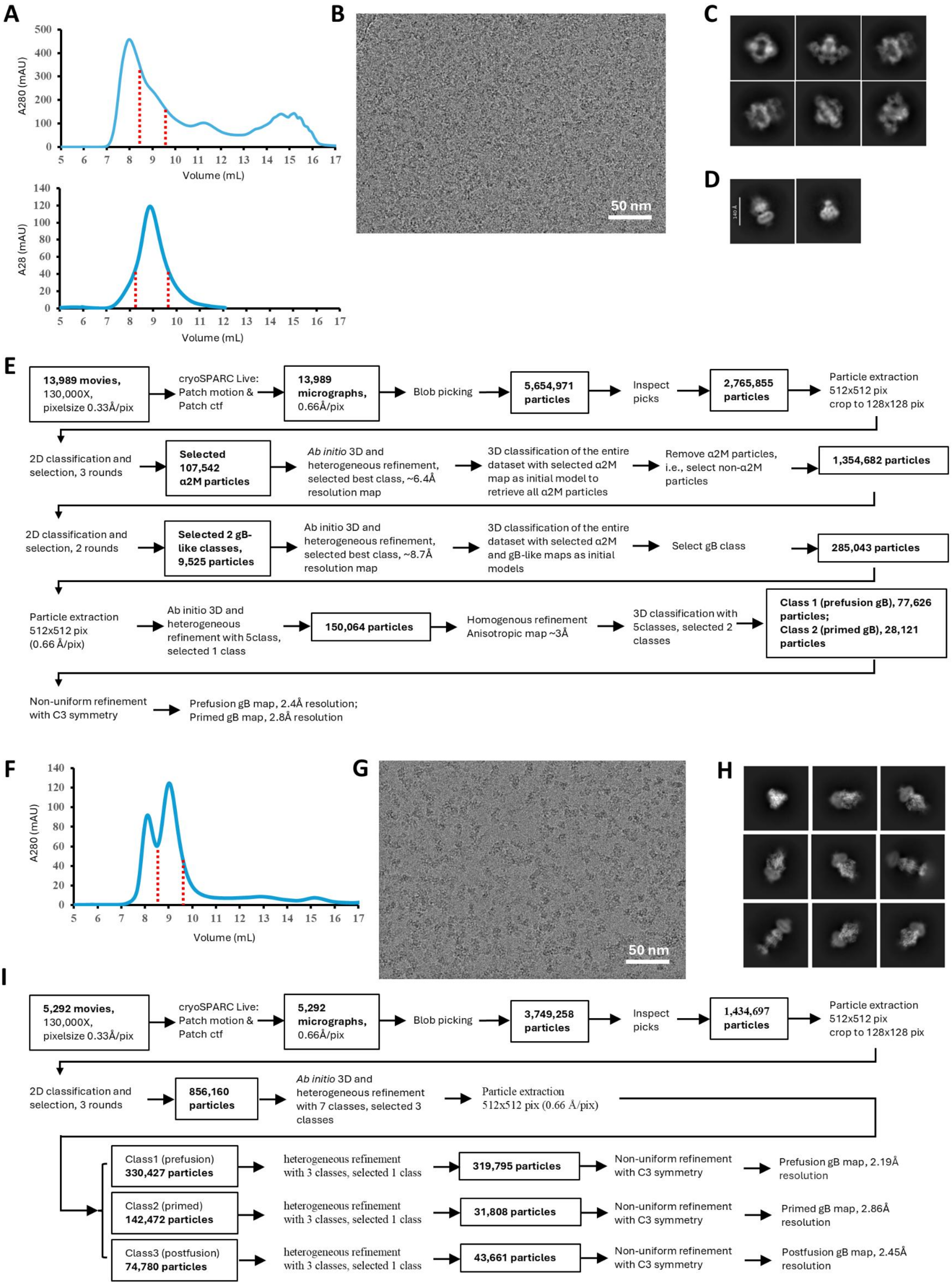
A
A280 (mAU)
Volume (mL)
A28 (mAU)
Volume (mL)
B
50 nm
C
D
E
13,989 movies,
130,000X,
pixelsize 0.33Å/pix
cryoSPARC Live:
Patch motion &
Patch ctf
13,989
micrographs,
0.66Å/pix
Blob picking
5,654,971
particles
Inspect
picks
2,765,855
particles
Particle extraction
512x512 pix
crop to 128x128 pix
2D classification and
selection, 3 rounds
Selected
107,542
α2M particles
Ab initio 3D and
heterogeneous refinement,
selected best class, ~6.4Å
resolution map
3D classification of the entire
dataset with selected α2M
map as initial model to
retrieve all α2M particles
Remove α2M particles,
i.e., select non-α2M
particles
1,354,682 particles
2D classification and
selection, 2 rounds
Selected 2 gB-
like classes,
9,525 particles
Ab initio 3D and
heterogeneous refinement,
selected best class, ~8.7Å
resolution map
3D classification of the entire
dataset with selected α2M
and gB-like maps as initial
models
Select gB class
285,043 particles
Particle extraction
512x512 pix
(0.66 Å/pix)
Ab initio 3D and
heterogeneous
refinement with 5class,
selected 1 class
150,064 particles
Homogenous refinement
Anisotropic map ~3Å
3D classification with
5classes, selected 2
classes
Class 1 (prefusion gB), 77,626
particles;
Class 2 (primed gB), 28,121
particles
Non-uniform refinement
with C3 symmetry
Prefusion gB map, 2.4Å resolution;
Primed gB map, 2.8Å resolution
F
A280 (mAU)
Volume (mL)
G
50 nm
H
I
5,292 movies,
130,000X,
pixelsize 0.33Å/pix
cryoSPARC Live:
Patch motion &
Patch ctf
5,292
micrographs,
0.66Å/pix
Blob picking
3,749,258
particles
Inspect
picks
1,434,697
particles
Particle extraction
512x512 pix
crop to 128x128 pix
2D classification and
selection, 3 rounds
856,160
particles
Ab initio 3D and
heterogeneous refinement
with 7 classes, selected 3
classes
Particle extraction
512x512 pix (0.66 Å/pix)
Class1 (prefusion)
330,427 particles
heterogeneous refinement
with 3 classes, selected 1 class
319,795 particles
Non-uniform refinement
with C3 symmetry
Prefusion gB map, 2.19Å
resolution
Class2 (primed)
142,472 particles
heterogeneous refinement
with 3 classes, selected 1 class
31,808 particles
Non-uniform refinement
with C3 symmetry
Primed gB map, 2.86Å
resolution
Class3 (postfusion)
74,780 particles
heterogeneous refinement
with 3 classes, selected 1 class
43,661 particles
Non-uniform refinement
with C3 symmetry
Postfusion gB map, 2.45Å
resolution

Figure S1. CryoEM sample preparation and data processing for virion-derived gB (A-E) or overexpressed gB (F-I). (**A**) Tandem size-exclusion chromatography (SEC) profiles of detergent-solubilized HSV-1 virion. The fractions between the two red dashed lines in the first run were pooled and loaded on to the second run. Fractions between the two red dashed lines in the second run were harvested for cryoEM sample freezing. (**B**) A representative cryoEM micrograph. (**C**) Representative 2D class averages of the dominating "contaminant"—alpha-2-macroglobulin (α2M). (**D**) 2D class averages of initially identified gB-like particles. (**E**) Data processing scheme leading to the determination of gB structures from the virion-derived sample. (**F**) SEC profile of overexpressed and affinity-purified HSV-1 gB. Fractions between the two red dashed lines were used for cryoEM. The first peak was in the void volume. (**G**) A representative cryoEM micrograph of the purified gB. (**H**) Representative 2D class averages. (**I**) Data processing scheme.

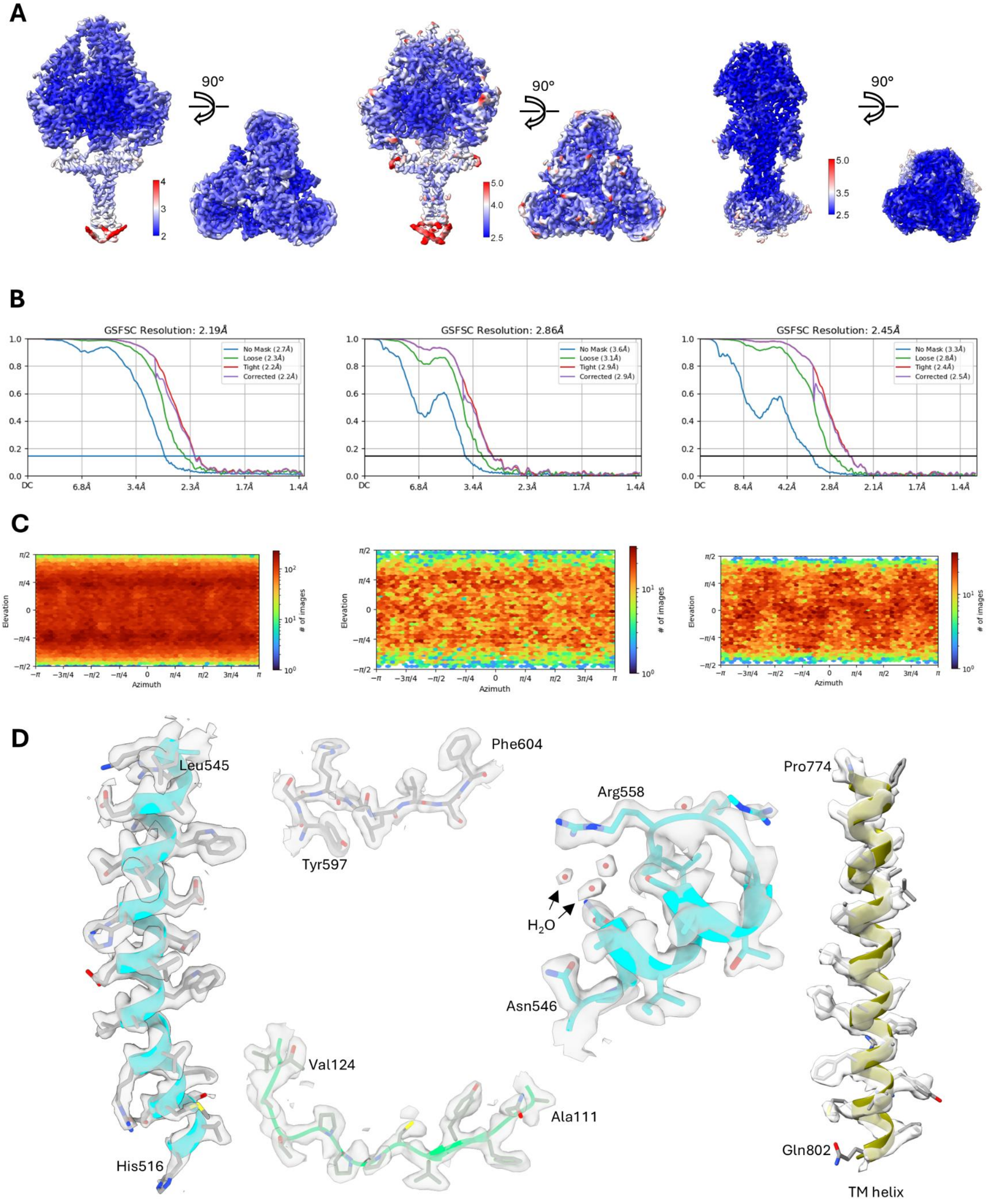
A
90°
B
GSFSC Resolution: 2.19Å
No Mask (2.7Å)
Loose (2.3Å)
Tight (2.2Å)
Corrected (2.2Å)
GSFSC Resolution: 2.86Å
No Mask (3.6Å)
Loose (3.1Å)
Tight (2.9Å)
Corrected (2.9Å)
GSFSC Resolution: 2.45Å
No Mask (3.3Å)
Loose (2.8Å)
Tight (2.4Å)
Corrected (2.5Å)
C
Elevation
Azimuth
# of images
D
Leu545
Phe604
Tyr597
Arg558
Pro774
H2O
Asn546
Val124
Ala111
His516
Gln802
TM helix

Figure S2. Quality assessment of the gB structures resolved from the overexpressed sample. (**A**) Local resolution of cryoEM density maps for HSV-1 gB prefusion state (left), primed state (middle) and postfusion state (right). Side and top views of each structure were surface colored as per local resolution values according to the scale bar presented in the middle. (**B**) Gold standard Fourier shell correlation (GSFSC) plot of the density maps shown in (A). The line in the bottom represents the 0.143 cutoff. (**C**) Orientation distribution plot for particles used in the final reconstruction of the density maps shown in (A). (**D**) Segmented density maps and their corresponding atomic models showcasing the high-resolution features of the cryoEM structure. The 2.19Å resolution map of the prefusion state was used. Only the N- and C-terminal residues of the segments were labeled for simplicity. Note that ordered water molecules (pointed to by arrows) were clearly resolved at this resolution.

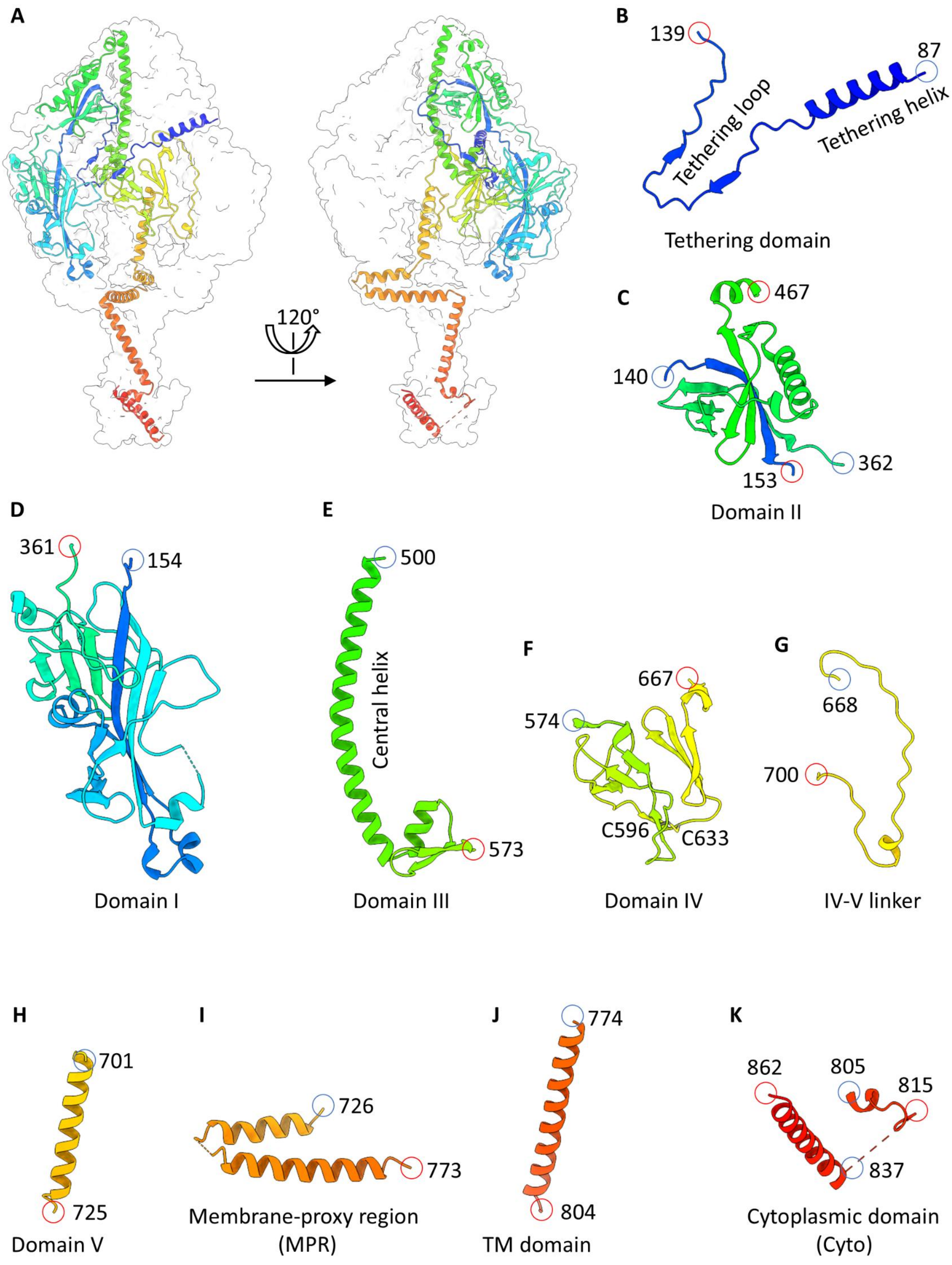

A
120°
B
139
87
Tethering loop
Tethering helix
Tethering domain
C
467
140
362
153
Domain II
D
361
154
Domain I
E
500
Central helix
573
Domain III
F
667
574
C596
C633
Domain IV
G
668
700
IV-V linker
H
701
725
Domain V
I
726
773
Membrane-proxy region
(MPR)
J
774
804
TM domain
K
862
805
815
837
Cytoplasmic domain
(Cyto)

Figure S3. Individual domains of HSV-1 gB in the prefusion state. (**A**) Atomic model of a protomer in the prefusion gB trimer rainbow-colored from blue (amino-terminus) to red (carboxyl-terminus). A white ghost of the whole complex is superimposed in the background to show the overall shape. (B-K) Structures of individual domains. Each domain is displayed in the same view as either the left panel or the right panel in (A). Domain segmentation is according to that defined in Fig. 2A. Terminal residues of the resolved regions are circled (blue for amino-terminus and red for carboxyl-terminus) and labeled. The intradomain disulfide bond in domain IV is also labeled (F). Note that domain II-III linker, residues 469-498, is completely flexible in the prefusion state and the primed state, and thus not shown here. A short segment in its N-terminal region becomes ordered (purple in Fig. 2H and Fig. 3G) by interacting with domain II in the postfusion state.

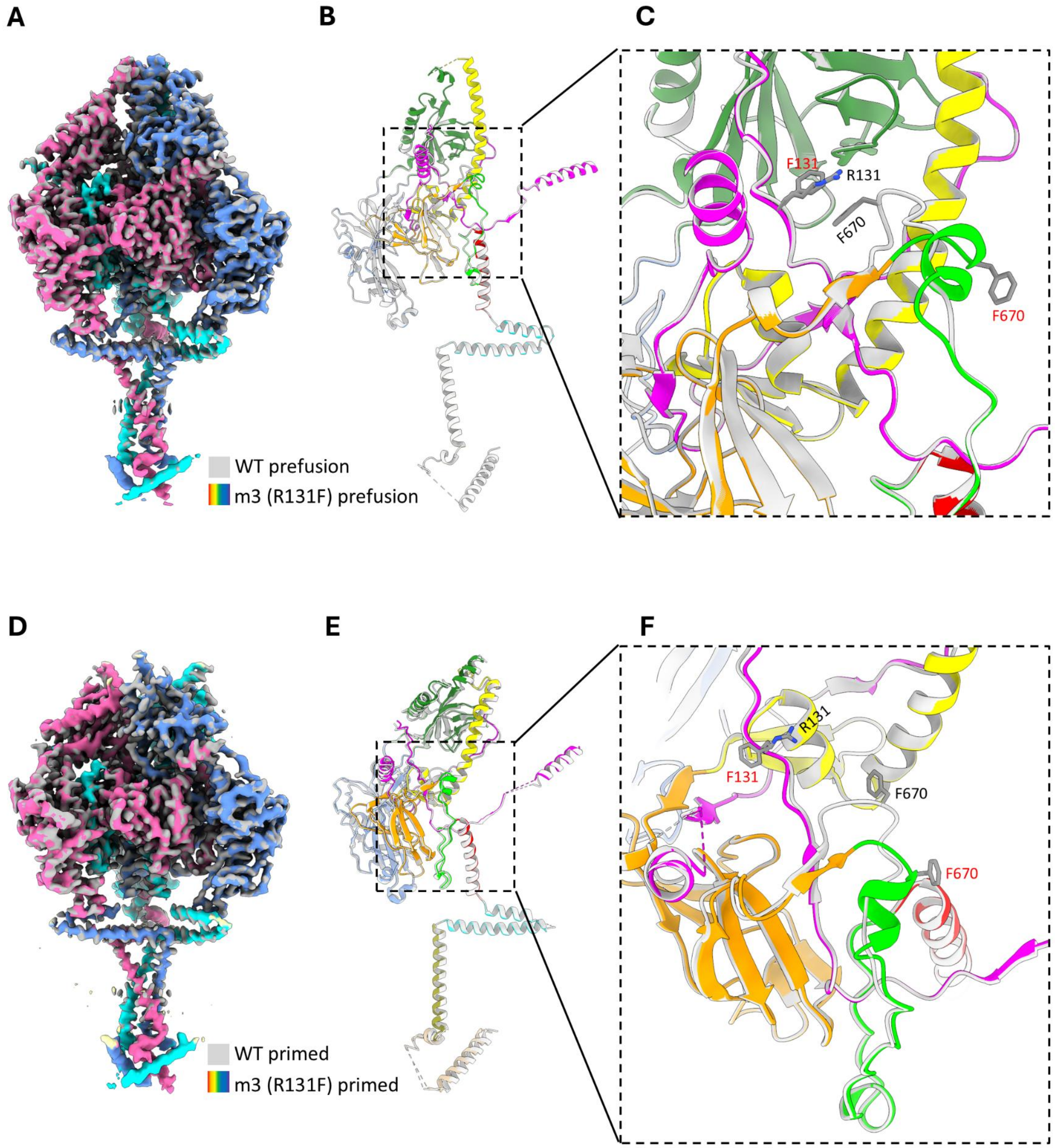

Figure S4. Comparing structures of the WT gB and mutant m3 (R131F) in the prefusion state (A-C) or the primed state (D-F). (**A**) and (**D**) Superposition of cryoEM density maps of WT gB and mutant m3 to show their overall similarity in the prefusion state (A) or the primed state (D). (**B**) and (**E**) Superposition of atomic models of WT gB and mutant m3 in the prefusion state (B) or the primed state (E). (**C**) and (**F**) Zoom-in views of the boxed regions in (B) and (E) to show the local structural changes around the mutation site in the prefusion state (C) or the primed state (F). Side chains in the WT structure are labeled in black, and those in the mutant are labeled in red. Note the stacking interaction between R131 and F670 in the WT prefusion structure (grey in C). As a result of the R131F mutation and loss of this stacking interaction, a segment of the IV-V linker in the mutant m3 (green in C and F) "retracts" and refolds into a short helix.

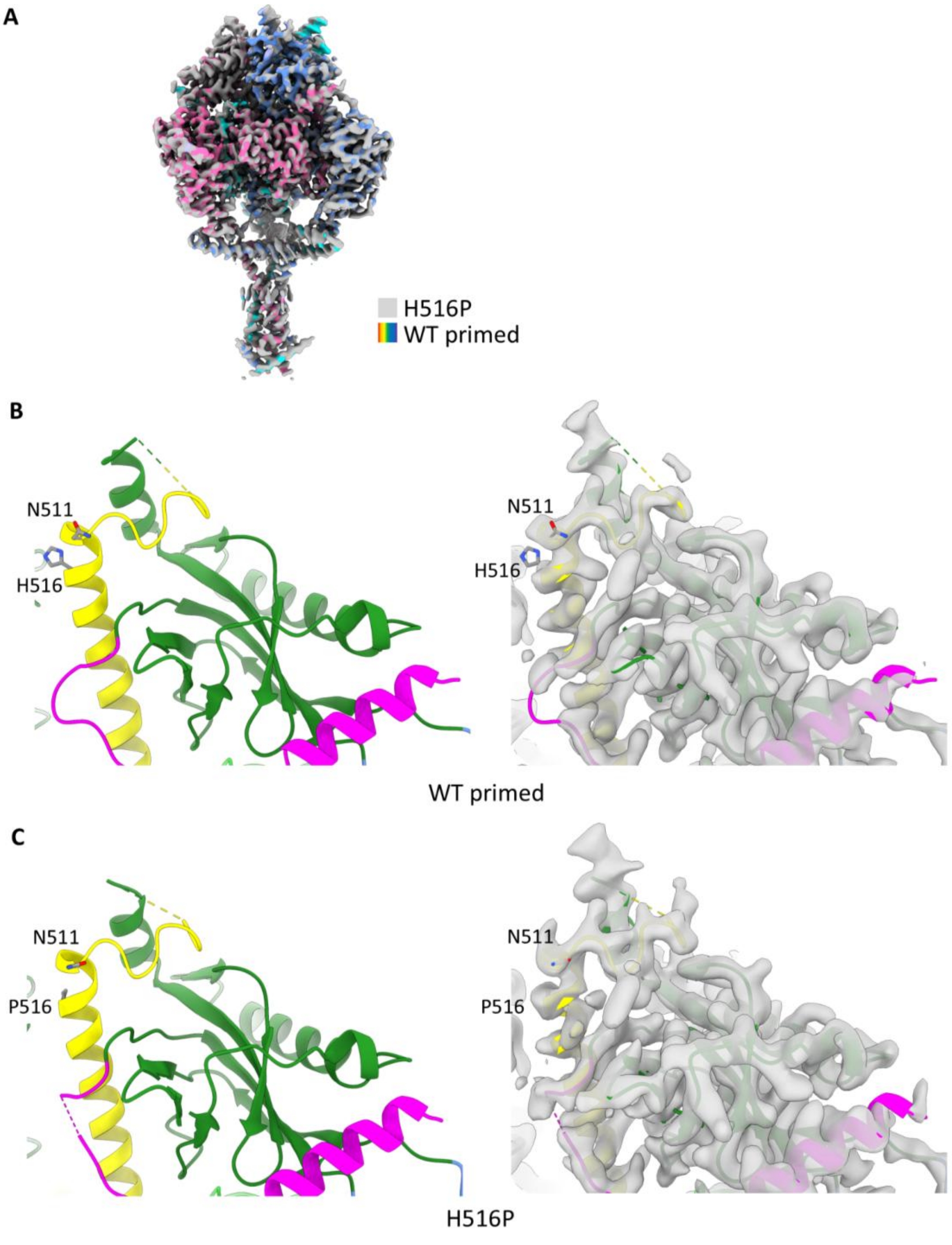

Figure S5. Structural characterization of the H516P mutant. (**A**) Superimposition of cryoEM density maps of HSV-1 gB WT (colored) and the H516P mutant (grey) at the primed state. (**B**, **C**) Side-by-side comparison of the atomic models of the WT (B) and the H516P mutant (C) around the mutation site. Semitransparent cryoEM density maps fitted with the corresponding atomic models are shown in the right. Note that the P516 residue in the H516P mutant retains an α-helical conformation, and the central helix still kinks around N511 as in the WT.

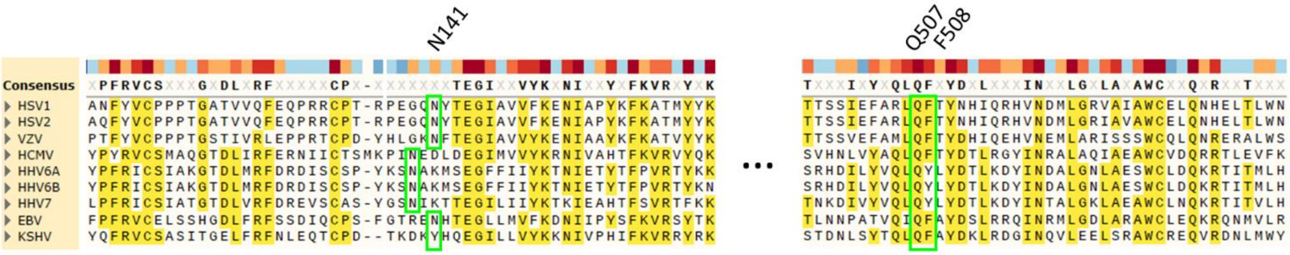

Figure S6. Multi-sequence alignment of all human herpesvirus gB, highlighting the domain II (left) and domain III central helix (right) regions. The green boxes highlight key residues corresponding to those mediating the stacking interactions between the folded tip of domain III central helix (Q507, F508) and domain II (N141) as shown in Fig 3I left panel. Conservation of these residues suggests that the priming mechanism we propose for HSV-1 gB may also apply to all other herpesviruses.

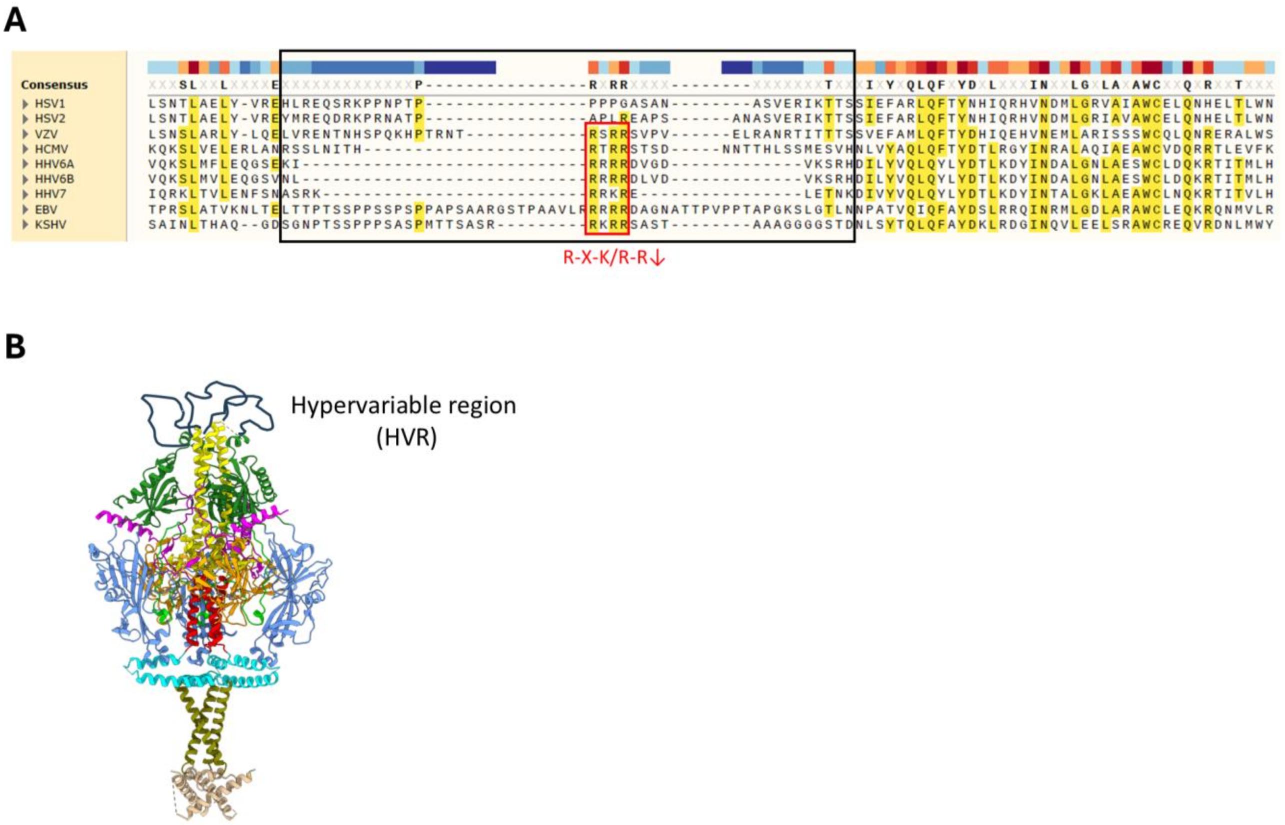

Figure S7. The hypervariable region (HVR) in gB sequences. (**A**) Multiple sequence alignment of all human herpesvirus gB, highlighting the hypervariable region (black box). This region is also named the II-III linker based on its localization in the gB structure, linking domain II to domain III. A furin cleavage site is present in this region for some gB (red box, with the furin recognition motif shown below). (**B**) A schematic drawing showing the highly flexible coils of the HVR mask the apex of the prefusion gB.

Table S1. Cryo-EM data collection, refinement and validation statistics.

	#1 HSV-1 gB WT prefusion (EMDB-47361, PDB 9E0L)	#2 HSV-1 gB WT primed (EMDB-47484, PDB 9E3I)	#3 HSV-1 gB WT postfusion (EMDB-47529, PDB 9E5V)	#4 HSV-1 gB mutant R131F prefusion (EMDB-47973, PDB 9EFH)	#5 HSV-1 gB mutant R131F primed (EMDB-47978, PDB 9EFS)	#6 HSV-1 gB mutant R131F deep-primed (EMDB-47988, PDB 9EGG)
**Data collection and processing**						
Magnification	130,000x	130,000x	130,000x	130,000x	130,000x	130,000x
Voltage (kV)	300	300	300	300	300	300
Electron exposure (e–/Å^2)	50	50	50	50	50	50
Defocus range (μm)	-0.8 to -1.5	-0.8 to -1.5	-0.8 to -1.5	-0.8 to -1.5	-0.8 to -1.5	-0.8 to -1.5
Pixel size (Å)	0.66	0.66	0.66	0.66	0.66	0.66
Symmetry imposed	C3	C3	C3	C3	C3	C3
Initial particle images (no.)	1,434,697	1,434,697	1,434,697	1,179,047	1,179,047	1,179,047
Final particle images (no.)	319,795	31,808	43,661	86071	87722	72160
Map resolution (Å)	2.19	2.86	2.45	2.49	2.62	2.65
FSC threshold	0.143	0.143	0.143	0.143	0.143	0.143
Map resolution range (Å)	2.1-4.0	2.8-4.0	2.4-4.0	2.4-4.0	2.6-4.0	2.6-4.0
**Refinement**						
Initial model used (PDB code)						
Model resolution (Å)	2.19	2.86	2.45	2.49	2.62	2.65
FSC threshold	0.143	0.143	0.143	0.143	0.143	0.143
Model resolution range (Å)	2.1-4.0	2.8-4.0	2.4-4.0	2.4-4.0	2.6-4.0	2.6-4.0
Map sharpening *B* factor (Å^2)	74.4	75.3	60.4	78.1	84.9	87.1
Model composition						
Non-hydrogen atoms	17476	16818	14319	17013	16143	11832
Protein residues	2142	2103	1770	2133	2019	1467
Ligands	6	6	3	6	6	6
B factors (Å^2)						
Protein	0.7/135/39.7	13.2/194/75.9	17.2/150/53	4/175/57.9	2.7/130/54.5	17.4/150/64
Ligand	21.3/74.3/55.7	71.3/115/96.4	63/84.8/74.2	46.9/105.4/86	70.5/116/96	63/124/92
R.m.s. deviations						
Bond lengths (Å)	0.003	0.004	0.004	0.002	0.003	0.004
Bond angles (°)	0.534	0.496	0.470	0.494	0.543	0.558
Validation						
MolProbity score	1.60	1.63	1.48	1.47	1.64	1.65
Clashscore	4.07	6.04	3.74	3.28	4.94	4.45
Poor rotamers (%)	2.62	2.44	2.6	2.86	2.71	3.67
Ramachandran plot						
Favored (%)	97.59	98.16	99.43	98.15	97.79	98.6
Allowed (%)	2.41	1.84	0.57	1.85	2.21	1.4
Disallowed (%)	0.00	0.00	0.00	0.00	0.00	0.00

	#7 HSV-1 gB mutant H516P primed (EMDB-47979, PDB 9EFT)	#8 HSV-1 gB mutant N511P primed (EMDB-47528, PDB 9E5U)	#9 HSV-1 gB mutant H534 primed (EMDB-47527, PDB 9E5N)	#10 HSV-1 gB mutant S392C, A527C prefusion (EMDB-47530, PDB 9E5X)	#11 HSV-1 gB mutant R131F, L97E, L101E postfusion (EMDB-47676, PDB 9E7I)
**Data collection and processing**					
Magnification	130,000x	130,000x	130,000x	130,000x	130,000x
Voltage (kV)	300	300	300	300	300
Electron exposure (e–/Å^2)	50	50	50	50	50
Defocus range (μm)	-0.8 to -1.5	-0.8 to -1.5	-0.8 to -1.5	-0.8 to -1.5	-0.8 to -1.5
Pixel size (Å)	0.66	0.66	0.66	0.66	0.66
Symmetry imposed	C3	C3	C3	C3	C3
Initial particle images (no.)	2,035,726	1,233,571	1,810,518	1,525,132	1,401,412
Final particle images (no.)	363,741	148,818	499,776	169,807	170,106
Map resolution (Å)	2.36	2.46	2.2	2.55	2.51
FSC threshold	0.143	0.143	0.143	0.143	0.143
Map resolution range (Å)	2.3-4.0	2.4-4.0	2.2-4.0	2.5-4.0	2.5-4.0
**Refinement**					
Initial model used (PDB code)					
Model resolution (Å)	2.36	2.46	2.2	2.55	2.51
FSC threshold	0.143	0.143	0.143	0.143	0.143
Model resolution range (Å)	2.3-4.0	2.3-4.0	2.2-4.0	2.5-4.0	2.5-4.0
Map sharpening *B* factor (Å^2)	87.1	83.2	81.6	91.0	91.2
Model composition					
Non-hydrogen atoms	16224	17043	16992	16230	11832
Protein residues	2031	2130	2097	2040	1467
Ligands	6	6	6	3	6
B factors (Å^2)					
Protein	0/31.9/6.2	2.7/156/50.7	3.8/172.6/66.4	4.1/146/55	6.6/116.0/39.8
Ligand	6.9/16.7/13.3	57/95/76	47.9/77.1/61.3	51/96/77	37.2/84.6/60.9
R.m.s. deviations					
Bond lengths (Å)	0.002	0.002	0.003	0.003	0.004
Bond angles (°)	0.455	0.471	0.547	0.578	0.594
Validation					
MolProbity score	1.46	1.63	1.6	1.53	1.83
Clashscore	3.98	4.02	4.33	4.16	4.32
Poor rotamers (%)	1.94	3.58	2.84	2.69	6.46
Ramachandran plot					
Favored (%)	97.75	97.91	97.82	98.96	98.74
Allowed (%)	2.25	2.09	2.18	1.04	1.26
Disallowed (%)	0.00	0.00	0.00	0.00	0.00
